# Compulsive sexual behavior may be more state-like than trait-like: Findings from a three-year longitudinal representative survey

**DOI:** 10.1556/2006.2025.00419

**Published:** 2026-04-15

**Authors:** Zsolt Horváth, Beáta Bőthe, Marc N. Potenza, Dan J. Stein, Süleyman Agah Demirgül, Borbála Paksi, Andrea Czakó, Zsolt Demetrovics

**Affiliations:** 1 Institute of Psychology, ELTE Eötvös Loránd University, Budapest, Hungary; 2 Centre of Excellence in Responsible Gaming, University of Gibraltar, Gibraltar, Gibraltar; 3 Département de Psychologie, Université de Montréal, Montréal, Canada; 4 Centre de recherche interdisciplinaire sur les problèmes conjugaux et les agressions sexuelles (CRIPCAS), Canada; 5 Department of Psychiatry, Yale School of Medicine, New Haven, CT, USA; 6 Child Study Center, Yale University School of Medicine, New Haven, CT 06510, USA; 7 Department of Neuroscience, Yale University, New Haven, CT 06510, USA; 8 Connecticut Mental Health Center, 34 Park Street, New Haven, CT 06519, USA; 9 Connecticut Council on Problem Gambling, Wethersfield, CT 06109, USA; 10 Wu Tsai Institute, Yale University, New Haven, CT 06510, USA; 11 SAMRC Unit on Risk & Resilience in Mental Disorders, Dept of Psychiatry & Neuroscience Institute, University of Cape Town, South Africa; 12 Doctoral School of Psychology, ELTE Eötvös Loránd University, Budapest, Hungary; 13 Institute of Education, ELTE Eötvös Loránd University, Budapest, Hungary; 14 Flinders University Institute for Mental Health and Wellbeing, College of Human Sciences and Culture, Flinders University, Bedford Park, SA, Australia

**Keywords:** addictive behaviors, behavioral addictions, compulsive behaviors, impulsive behaviors, compulsive sexual behavior disorder, hypersexuality, latent state-trait theory, sexual addiction, sexual behavior

## Abstract

**Background and aims:**

Studies regarding whether compulsive sexual behavior (CSB) shows a persistent or transient longitudinal course have been scarce and generated mixed findings, complicating its conceptualization. A model that includes both trait-like/stable and state-like/occasion-specific factors may clarify the course and nature of CSB. The authors aimed to determine the proportion of variance in CSB scores attributable to trait-like/stable and state-like/occasion-specific factors and examine the strength of autoregressive effects between successive state-like/occasion-specific factors.

**Methods:**

Data from young adults (*N* = 782; women: 39.60% [*N* = 310]; age at wave 1: *M* = 27.06 [*SD* = 4.69]) showing continuous sexual activity from waves 1–4 of the representative Budapest Longitudinal Study were analyzed. CSB was measured with the eight-item Hypersexual Behavior Inventory.

**Results:**

Latent growth modeling showed decreases in CSB over waves 1–4 across three years, following a non-linear (quadratic) trajectory (*M* [*SE*] = −0.26 [0.08]). A latent state-trait model with one CSB trait-like/stable factor and four state-like/occasion-specific residual factors (capturing time-specific fluctuations) had optimal fit (*χ2* [*df*] = 653.667 [550], *p* = 0.002; *CFI* = 0.996; *TLI* = 0.997; *RMSEA* [90% *CI*] = 0.016 [0.011; 0.021]; *SRMR* = 0.075). High proportions of CSB score variance were attributable to state-like/occasion-specific factors (69–93%), while lower proportions of CSB scores were attributable to the trait-like/stable factor (7–31%). Additionally, positive, moderate-to-strong autoregressive effects were observed between successive state-like/occasion-specific factors, indicating short-term stability and carry-over across assessments (*β* [*SE*] = 0.36–0.65 [0.06–0.08]).

**Discussion and conclusions:**

Overall, CSB is more likely to be a state-like phenomenon in young adults from the general population. Situational influences may explain the longitudinally fluctuating and transient course of CSB. Significant state-like/occasion-specific effects may have important implications for screening for and diagnosis of CSB disorder.

## Introduction

### The longitudinal course of compulsive sexual behavior

In the 11th revision of the International Classification of Diseases (ICD-11), compulsive sexual behavior disorder (CSBD) is classified as an impulse control disorder and defined by poor control over repetitive sexual urges and behaviors and continued behavior despite negative consequences and/or significant psychological distress (World Health Organization, 2019). Additional factors not included in the diagnostic criteria may have clinical relevance, including the use of sex to cope with negative affective states (Kafka, 2010; World Health Organization, 2019; Ince et al., 2026). However, these symptoms are not limited to people with clinical disorders. A considerable proportion of the general population may show compulsive sexual behavior (CSB) and be at risk for CSBD (Bőthe et al., 2023). However, it is important to acknowledge that various terms appear in the literature to describe these constructs, including hypersexuality, sexual addiction, and sexual compulsivity (Bőthe et al., 2023; Brand et al., 2025). To maintain consistency, the present study reserves CSBD for the diagnostic condition defined by the ICD-11, while CSB refers to symptom severity and consequences resulting from problematic sexual behavior, without systematically assessing a clinical diagnosis.

Limited and inconclusive evidence exists regarding the longitudinal course of CSB. As part of the present study, a brief systematic review was conducted to identify and synthesize observational studies examining longitudinal patterns of CSB (Supplementary Fig. 1; Supplementary Tables 1 and 2). Relevant articles were identified based on the most recent systematic review (Grubbs, Hoagland, et al., 2020), supplemented by an additional PubMed literature search targeting studies published subsequently (Supplementary Fig. 1 and Supplementary Table 1). Altogether, 12 observational studies (published between 2012 and 2024) were identified that provided statistical data on the longitudinal course of CSB (Supplementary Table 2). Overall, these studies demonstrated heterogeneous patterns. First, several studies indicated longitudinal stability in CSB, supported by moderate-to-high correlations between consecutive CSB scores and negligible-to-small changes in CSB levels over time (Ballester-Arnal, Gómez-Martínez, Llario, & Salmerón-Sánchez, 2013; Bőthe, Vaillancourt-Morel, & Bergeron, 2021; Efrati, 2024; Gomez, Brown, & Stavropoulos, 2024; Reid et al., 2012; Rosansky, Borgogna, Kraus, & Grubbs, 2022). Although this pattern was found predominantly in non-clinical samples, this pattern may parallel the symptom trajectories of treatment-seeking individuals who report persistent CSB severity without remission (Reid et al., 2012). Second, multiple studies reported significant or strong decreases in CSB severity over time (Deng, Li, Wang, & Teng, 2021; Grassi, Moradei, & Cecchelli, 2024; Smith et al., 2014; Thompson, Kingree, Zinzow, & Swartout, 2015). Among non-clinical samples, this pattern may reflect natural, non-therapeutic recovery processes similar to those documented for certain potentially addictive behaviors (Konkolÿ Thege, Woodin, Hodgins, & Williams, 2015). Third, some studies observed increases in CSB severity across particular periods (e.g., COVID-19 pandemic and related lockdowns); however, these changes were generally temporary and negligible (Koós, Demetrovics, Griffiths, & Bőthe, 2022; Kürbitz et al., 2022). These fluctuations may reflect unstable and transient manifestations of CSB symptoms over time (Castro-Calvo, Ballester-Arnal, Giménez-García, García-Barba, & Gil-Llario, 2023). Extending this third pattern, it is important to acknowledge that some treatment-seeking individuals may experience a gradual progression in CSB severity, further highlighting the possible heterogeneity of longitudinal trajectories (Reid et al., 2012).

### Stable and situational characteristics of compulsive sexual behavior

A novel perspective on understanding the longitudinal course of CSB may be obtained by considering a hybrid longitudinal structure (Geiser, 2021; Geiser, Hintz, Burns, & Servera, 2021). Building on the latent state-trait (LST) theory, this model suggests that the variance of true CSB symptom scores over time may be influenced by both trait-like/stable and state-like/occasion-specific factors (Geiser, 2021; Geiser et al., 2021). In practical terms, the severity of CSB observed at a particular time point arises from the joint contribution of trait-like/stable and state-like/occasion-specific influences (Conway, Hopwood, Morey, & Skodol, 2018). In contrast, temporal stability as reflected by traditional test–retest correlations and autoregressive effects in a cross-lagged panel model cannot specifically assess trait-like/stable and state-like/occasion-specific influences, which is essential in longitudinal measurement contexts. In other words, these traditional longitudinal metrics cannot clarify whether the observed stability reflects a genuine stable disposition or temporary situational effects. These indices implicitly assume that individuals fluctuate around the same time-specific means, thereby ignoring person-specific stable levels that reflect enduring trait-like characteristics (Hamaker, Kuiper, & Grasman, 2015). The use of an LST-based approach is further supported by findings showing that trait-like/stable and state-like/occasion-specific longitudinal structures also characterize other psychopathologies (Conway et al., 2018; Horváth et al., 2024).

A trait-like/stable factor of CSB represents a temporally stable/invariant construct, which indicates a general/constant proneness across measurement points. Thus, the trait-like/stable factor may represent an individual's temporally unchanging and habitual level of CSB severity, along which between-person differences can be described, and around which within-person fluctuations occur across measurement occasions (Geiser, 2021; Geiser et al., 2021). It can be assumed that an individual's temporally stable, habitual level of CSB may be shaped by factors such as stable personality characteristics (e.g., higher neuroticism, impulsivity, and difficulties in emotion regulation, lower agreeableness), early life experiences (e.g., insecure attachment patterns, childhood sexual abuse), and neurocognitive mechanisms (e.g., sensitization, habituation, impulse dysregulation, and reward processing) (Brazil, 2025; Kowalewska et al., 2018; Lew-Starowicz, Lewczuk, Nowakowska, Kraus, & Gola, 2020; Slavin, Scoglio, Blycker, Potenza, & Kraus, 2020).

A state-like/occasion-specific factor of CSB reflects a temporally unstable/varying construct, representing an occasion-specific situational influence on CSB at a given measurement point (Geiser, 2021; Geiser et al., 2021). Examples of such state-like/occasion-specific influences may include negative life events, acute stress, interpersonal conflict, changes in romantic relationship status, transient affective states (e.g., loneliness, anxiety, shame, or guilt or regret related to sexual behavior), fluctuations in sexual desire, increased access to sexualized online content, or exposure to high-risk situations (e.g., alcohol or drug use) that may momentarily heighten CSB severity (Grant Weinandy, Lee, Hoagland, Grubbs, & Bőthe, 2023; Mestre-Bach & Potenza, 2023; Wizła & Lewczuk, 2024).

In addition to trait-like/stable and state-like/occasion-specific influences, autoregressive effects are also relevant for understanding the longitudinal course of CSB. In LST-based models, these autoregressive paths are specified between consecutive state-like/occasion-specific factors, representing carry-over effects from one measurement occasion to the subsequent one. Accordingly, autoregressive effects capture short-term temporal stability over and above the influence of the trait-like/stable factor (Geiser et al., 2021). For example, experiencing acute stress (e.g., work-related stress or a relationship breakup) may temporarily elevate CSB above a person's habitual level, and this temporary increase may carry over to the subsequent measurement occasion. It is expected that stronger autoregressive effects could be observed, for example, among individuals who reported a gradual progression in severity of CSB over time (Reid et al., 2012). In addition, autoregressive effects could be relevant and harmonized with theoretical approaches explaining the clinical course and stages of CSBD through its motivational background (i.e., shift from positive to negative reinforcement as progressing to more severe CSBD) (Antons & Brand, 2021; Briken & Turner, 2022).

Overall, if CSB is a more trait-like/stable construct, then smaller intra-individual variations in the severity of CSB over time are expected around the temporally-stable/trait level of CSB (Geiser et al., 2021). This longitudinal pattern may be comparable to continuous and stable symptomatic CSB courses (Reid et al., 2012). By contrast, if CSB appears to be a more state-like/occasion-specific construct, greater fluctuations in CSB severity may be expected around the temporally-stable/trait CSB level due to situational influences and/or person-situation interactions (e.g., adverse life events) (Geiser et al., 2021). In line with this notion, a higher degree of instability and temporal variability of CSB was observed among individuals who had less severe CSB symptoms and were younger (Castro-Calvo et al., 2023).

In addition, the distinction between trait-like/stable and state-like/occasion-specific characteristics may offer further insights into the conceptualization of CSBD (Bőthe, Koós, & Demetrovics, 2022; Sassover & Weinstein, 2020). Although CSBD is listed among the impulse control disorders in the ICD-11 (World Health Organization, 2019), currently there is no consensus on whether CSBD should be best classified as an addictive, impulsive, or obsessive-compulsive-spectrum disorder (Bőthe et al., 2022; Sassover & Weinstein, 2020). For example, if CSB appears more state-like/occasion-specific, it may align more closely with an impulse control disorder, whereas a more trait-like/stable presentation would be more consistent with characteristics and chronic persistence of addictive or obsessive-compulsive disorders (Le Moal & Koob, 2007).

### The present study

Building on this background, the present study had two main aims:

1. To estimate the proportion of variance in CSB scores attributable to trait-like/stable versus state-like/occasion-specific influences.
2. To examine the strength of autoregressive effects between successive state-like/occasion-specific factors across time.

To address these aims, an LST modelling framework was applied. This represents the first implementation of this approach to investigate whether CSB is better conceptualized as a trait-like or state-like construct. This approach may contribute to a more nuanced understanding of the longitudinal course of CSB (Grubbs, Hoagland, et al., 2020).

## Methods

### Procedures

The present study was part of the Budapest Longitudinal Study (BLS) conducted between 2019 and 2022 (Horváth et al., 2023, 2024; Paksi et al., 2021). Longitudinal panel data from four successive waves were analyzed with approximately one-year intervals between each wave. The BLS targeted young adults (i.e., 18–34 years of age at study onset) living in Budapest, the capital city of Hungary. Accordingly, the sampling frame comprised individuals born between 1984 and 2000 who had an officially registered residential address in Budapest on 1 January 2019, as recorded in the national population registry. In total, the sampling frame included 321,974 individuals. To ensure that the sample reflected the demographic structure of this population, a one-stage random sampling procedure stratified by age group (two categories: 18–24 and 25–34 years) and by city district (23 districts) was implemented. In wave 1, individuals included in the gross sample were randomly selected from the population registry. Subsequent waves derived their gross samples from respondents of earlier waves. In all waves, individuals were contacted through postal invitation letters followed by repeated in-person attempts. Successful involvement rates were 86, 72, 77, and 93% in waves 1–4, respectively. Attrition occurred due to unavailability (e.g., unknown new address, death), permanent refusal, or failure to complete the online questionnaire when applicable. To address potential sample distortion due to attrition, multidimensional longitudinal weighting was employed in the analyses (see the Statistical analyses subsection for further details). Following data quality checks, additional participants were excluded from the final net samples. Data were primarily collected through in-person, interviewer-administered assessments (e.g., questions regarding socio-demographic information and the frequency of potentially addictive behaviors) combined with standardized self-report questionnaires (e.g., scales on addictive behaviors and psychological characteristics). In waves 2 and 3, participants could alternatively complete an online interview and questionnaire to reduce potential reluctance to participate during the COVID-19 pandemic, although only a very small proportion opted for the online format (*N* = 57 in wave 2 and *N* = 7 in wave 3) (Supplementary Fig. 2). The study was not pre-registered.

### Participants

In the BLS, 3,914 people participated, of whom 2,272 participated in all four waves (women: *N* = 1,159 [51.01%]; mean age at wave 1: 27.10 years [SD = 4.78]). The final sample involved those who (i) participated in all four waves, (ii) reported having sex, masturbating, or watching pornography at least once in the past year in all four waves, and (iii) had a valid response on at least one item of the short version of the Hypersexual Behavior Inventory (HBI-8) in all four waves. The second inclusion criterion was primarily defined to minimize potential confounding effects in the interpretation of HBI-8 scores. Including participants who reported no sexual activity (i.e., partnered sex, masturbation, or pornography use) in a given wave would have led to uncertainty concerning the meaning of “Never” responses on the HBI-8. Specifically, it would not have been possible to determine whether such responses reflected the absence of sexual activity (e.g., the lack of opportunities to experience CSB symptoms among sexually abstinent individuals) or the genuine absence of CSB symptoms (e.g., low CSB despite the presence of sexual activity). Restricting the sample to individuals with continuous sexual activity therefore ensured that score fluctuations reflect actual symptom dynamics rather than changes in opportunity for sexual behaviour. This approach may have contributed to a more precise differentiation between trait-like and state-like factors underlying longitudinal variation in CSB symptom scores and minimized artefactual score fluctuations. In addition, a third inclusion criterion was applied due to substantial missing values on HBI-8 items in wave 3 (*N* = 808–813). While the cause of this potentially systematic missing data pattern remained unclear, sensitivity analyses assessed the impact of the third inclusion criterion. Ultimately, 782 individuals were included in the final sample (women: *N* = 310 [39.60%]; mean age at wave 1: 27.06 years [*SD* = 4.69]).

### Measures

CSB was measured with the shortened HBI-8 (Bőthe et al., 2019; Reid, Garos, & Carpenter, 2011). Responses on the HBI-8 were provided on a five-point scale (1 = Never, 5 = Very often). The use of the HBI-8 in the BLS was supported by its ability to provide a time-efficient and valid assessment of CSB, as it showed a very strong correlation with the full 19-item version of the HBI (HBI-19) (Bőthe et al., 2019). Similar to the HBI-19, it allows for the calculation of a total score reflecting overall CSB severity, as well as specific subscale scores for consequences (e.g., *“I sacrifice things I really want in life in order to be sexual.”*), control (e.g., *“Even though my sexual behavior is irresponsible or reckless, I find it difficult to stop.”*), and coping (e.g., *“When I feel restless, I turn to sex in order to soothe myself.”*). Higher total and subscale scores indicate more pronounced manifestations of CSB, both globally and within the specific dimensions assessed. To ensure more parsimonious analyses, only the total scale score was used in the present non-LST model-based analyses. The HBI-8 demonstrated high internal reliability across all study waves, with Cronbach's alpha coefficients ranging between 0.92 and 0.96 (Table 1).

**Table 1.** Descriptive statistics and pairwise correlations between Hypersexual Behavior Inventory (HBI-8) scores in Waves 1–4 (W1–W4)

	1.	2.	3.	4.
1. W1 HBI-8 total score	–			
2. W2 HBI-8 total score	0.40***	–		
3. W3 HBI-8 total score	0.31***	0.55***	–	
4. W4 HBI-8 total score	0.03	0.14**	0.26***	–
*M* (SD)	10.48 (5.24)	10.40 (5.21)	9.88 (4.22)	8.76 (2.53)
Min – Max	8–40	8–40	8–36	8–33
Cronbach's *α*	0.96	0.94	0.92	0.93

*Notes:* W1-4: scores from wave 1–4, respectively. *M* (*SD*): mean (standard deviation). Min – Max: minimum and maximum values. Level of significance: *p < 0.05; **p < 0.01; ***p < 0.001.

### Statistical analyses

As a preliminary analysis, pairwise correlations were calculated between HBI-8 total scores across all waves. To examine linear and quadratic within-person changes between waves 1 and 4, latent growth modeling was performed based on total scores of the HBI-8. In line with previous studies, within-person changes were examined using the higher-order quadratic slope, whereas the linear slope was not considered (Cohen, Cohen, West, & Aiken, 2003).

Before testing the LST models, longitudinal measurement invariance analyses were performed for the HBI-8 to ensure equivalent measurement longitudinally. Based on prior findings (Bőthe et al., 2019), we tested a one-factor and a three-factor model (M1 and M2). First, model fit of each model was assessed separately in each wave. Next, the four increasingly restrictive levels of configural, metric, scalar, and residual invariance (Supplementary Table 3) were examined and compared (Liu et al., 2017).

The LST models (M3) were defined according to the best-fitting and most parsimonious models, with statistical parameters being defined following the accepted invariance levels (Geiser et al., 2021). The fit of four LST models was tested and compared (Supplementary Fig. 3), through which both Aim 1 and Aim 2 were addressed. These models included one trait-like/stable factor without specific method factors (M3a), one trait-like/stable factor and seven correlated method factors (M3b), and eight correlated indicator-specific trait-like/stable factors (M3c) in addition to the four state-like/occasion-specific residual factors (Geiser & Lockhart, 2012). M3a–M3c did not include correlation or autoregressive effects between state-like/occasion-specific residual factors. However, to specifically examine Aim 2, an alternative model that included autoregressive effects was also tested for the best-fitting LST model (M3d) (Geiser et al., 2021). Standardized occasion-specificity and consistency estimates were calculated to quantify the proportions of the observed variances of each true score on the HBI-8 that represents state-like/occasion-specific or trait-like/stable effects. These estimates were used to address Aim 1. Reliability coefficients were calculated to measure how much of the variance in observed scores expressed true score variance (and was not related to measurement error) (Geiser, 2021).

Pairwise correlations and latent growth modeling were estimated using the maximum likelihood robust to non-normality (MLR) procedure. Models based on M1 and M2 were tested using the weighted least squares means and variances adjusted (WLSMV) estimation procedure. The goodness-of-fit of these models was tested using the comparative fit index (*CFI*), Tucker-Lewis index (*TLI*), root mean square error of approximation (*RMSEA*) and standardized root mean square residual (*SRMR*). For the *CFI* and *TLI* > 0.900 and >0.950, and for the *RMSEA* and *SRMR* < 0.080 and <0.050 indicated adequate and optimal model fit, respectively.

Sensitivity analyses examined how the exclusion of participants with missing HBI-8 values influenced findings. The final sample was compared with excluded participants on sociodemographic factors and HBI-8 total scores. Additionally, preliminary analyses, longitudinal measurement invariance, and LST model testing for the best-fitting model were conducted on an alternative sample (*N* = 1,634; women: *N* = 787 [48.17%]; mean age at wave 1: 27.12 years [*SD* = 4.68]). Participants were eligible for sensitivity analyses if they (i) participated in all four waves and (ii) reported engaging in sex, masturbation, or pornography use at least once in the past year in each wave. However, the inclusion criterion for valid HBI-8 responses was not applied. These analyses included HBI-8 scores from waves 1, 2, and 4, while wave 3 data were excluded as missing values in wave 3 were assumed to follow a non-random/systematic pattern.

All analyses were performed in MPlus 8.0 using longitudinal weights (Muthén & Muthén, 2017). The multidimensional longitudinal weighting of the study accounted for the multivariate relationship between sample attrition and socio-demographic characteristics, in addition to adjusting for proportional population distributions by district of residence and age group. Predictors of attrition were examined using a binary logistic regression model, in which participation in all four waves was modeled as a function of the stratification dimensions and baseline socio-demographic characteristics. Gender, expected educational attainment, and religiosity emerged as significant predictors of retention across waves. Accordingly, attrition correction was implemented in two steps. First, raw weights were constructed using matrix weighting based on the joint distribution of the three variables significantly associated with retention, thereby adjusting the sample of individuals participating in waves 1–3 to conform to the distribution observed in the baseline sample. In the final step, to correct for attrition across all four waves, cell-preserving matrix calibration weighting was applied to align the weighted sample with the stratified population distribution of the baseline sample. Missing values were handled by the full information maximum likelihood (FIML) method in all analyses (Muthén & Muthén, 2017).

### Ethics

Participation was voluntary, and participants provided written informed consent. The authors assert that all procedures contributing to this work comply with the ethical standards of the relevant national and institutional committees on human experimentation and with the Helsinki Declaration of 1975, as revised in 2013. The study obtained ethical approval from the Research and Ethical Committee of the Medical Research Council (no. 60471–2/2018/EKU).

## Results

### Preliminary analyses

CSB scores correlated significantly and positively across consecutive waves (*r* = 0.26–0.55) (Table 1). Correlation strength varied across waves: moderate between waves 1–2, approaching the strong range between waves 2–3, and small between waves 3–4. A significant and negative quadratic within-person decrease in CSB was observed between waves 1 and 4 (Table 2). There were negligible changes in CSB across waves 1 through 3 (*d* = 0.01–0.12), whereas small decreases were observed at wave 4 compared with the preceding waves (*d* = 0.21–0.23; see Table 2 and Supplementary Fig. 4).

**Table 2.** Latent growth modelling based on the total score of the Hypersexual Behavior Inventory (HBI-8)

	Intercept	Linear slope	Quadratic slope	Correlation between intercept and linear slope	Correlation between intercept and quadratic slope	Correlation between linear and quadratic slopes
*M* (*SE*)	10.46 (0.23)***	0.22 (0.26)	−0.26 (0.08)**	–	–	–
*σ*^*2*^ (*SE*)	12.74 (4.28)**	11.89 (5.09)*	1.33 (0.50)**	–	–	–
*r* (*SE*)	–	–	–	−0.05 (0.27)	−0.29 (0.24)	−0.93 (0.03)***
Effect size for changes across waves (*d*)						
	Wave 1	Wave 2	Wave 3			
Wave 1	–	–	–			
Wave 2	0.01	–	–			
Wave 3	0.09	0.12	–			
Wave 4	0.22	0.23	0.21			

*Notes: M* (*SE*): mean (standard error). *σ*^*2*^ (*SE*): variance (standard error). *r* (*SE*): standardized covariance (standard error). *d*: repeated measures and pooled Cohen's *d* controlling for the intercorrelation across measurements. Level of significance: *p < 0.050; **p < 0.010; ***p < 0.001. Level of model fit: *χ*^*2*^ (1) = 0.136; *p* = 0.713; *RMSEA* [90% *CI*] = 0.000 [0.000–0.072]; *CFI* = 1.000; *TLI* = 1.000; *SRMR* = 0.003.

### Measurement invariance testing

The one-factor model (M1) demonstrated adequate and optimal fit across all four wave-specific models (M1a–M1d: *CFI* = 0.997–0.999, *TLI* = 0.995–0.999, *RMSEA* = 0.021–0.061, *SRMR* = 0.016–0.024), as well as across all four invariance models (M1e–M1h: *CFI* = 0.997–0.998, *TLI* = 0.997–0.998, *RMSEA* = 0.013–0.015, *SRMR* = 0.059–0.062; Supplementary Table 4). However, the three-factor model (M2) was not interpreted because the latent variable covariance matrix included out-of-range correlations. In several cases, correlations between latent factors exceeded 1.00, resulting in a non–positive definite covariance matrix. This indicated a non-admissible and unreliable factor solution (Supplementary Table 4). For M1, only a slight decrease in model fit (***Δ****CFI* = 0.000–0.001, ***Δ****TLI* = 0.000–0.001, ***Δ****RMSEA* = 0.000–0.002, ***Δ****SRMR* = 0.000–0.002) between the consecutive invariance levels was observed (Supplementary Tables 3 and 4). Thus, residual invariance level (M1h: *χ2* (548) = 635.755, *p* = 0.006; *CFI* = 0.997; *TLI* = 0.997; *RMSEA* [90% *CI*] = 0.015 [0.009; 0.020]; *SRMR* = 0.062) was reached (Supplementary Table 4). Significant, positive, and strong factor loadings were observed across all four wave-specific one-factor models (M1–M1d: *λ* = 0.76–0.97), as well as in the residual invariance model (M1h: *λ* = 0.87–0.96). In line with this, very high levels of internal reliability (McDonald's *ω* = 0.97–0.98) were shown for each factor in these models (Supplementary Table 5). In the residual invariance model, significant, positive, and strong inter-factor correlations (*r =* 0.51–0.70) were identified between consecutive wave-specific CSB factors, as well as between waves 1 and 3 (Supplementary Table 5).

### LST models

The LST models (M3a-M3d) were defined based on M1 (Supplementary Table 4). The model with one trait-like/stable factor and autoregressive effects (M3d: *χ2* (550) = 653.667, *p* = 0.002; *CFI* = 0.996; *TLI* = 0.997; *RMSEA* [90% *CI*] = 0.016 [0.011; 0.021]; *SRMR* = 0.075) showed adequate to optimal model fit. By contrast, in the model without autoregressive effects (M3a: *χ2* (553) = 785.245, *p* < 0.001; *CFI* = 0.991; *TLI* = 0.992; *RMSEA* [90% *CI*] = 0.024 [0.020; 0.028]; *SRMR* = 0.102), the CFI, TLI, and RMSEA indices indicated optimal fit, whereas the SRMR suggested inadequate fit. Overall, the model including autoregressive effects (M3d) demonstrated a consistently closer fit to the data (***Δ****χ2* (3) = 27.582, *p* < 0.001; ***Δ****CFI* = 0.005; ***Δ****TLI* = 0.005; ***Δ****RMSEA* = 0.008; ***Δ****SRMR* = 0.027) across all indicators than the model without autoregressive effects (M3a; Supplementary Table 4). Considering both statistical criteria and the conceptual relevance of autoregressive effects in the longitudinal course of CSB (see Introduction), the model with autoregressive effects (M3d) was retained and interpreted.

**Fig. 1.**
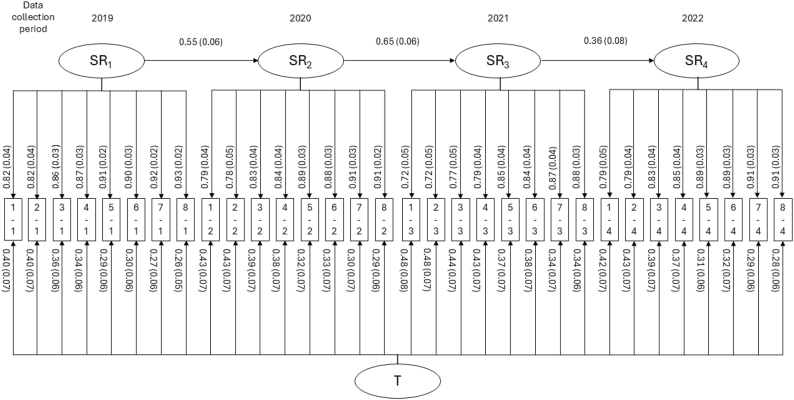
Latent state-trait (LST) model with one trait factor. Notes. Single-headed arrows between observed variables (in squares) and latent factors (in ellipse): standardized factor loadings (standard error). Single-headed arrows between latent factors: standardized regression coefficients (standard error). SR_1_–SR_4_: latent state residual factors. T: trait factor. Values related to each observed variable: The upper value represents the item number in the Hypersexual Behavior Inventory (see Table 3), while the lower value indicates the measurement wave. All factor loadings and regression coefficients are significant at *p* ≤ .001 level

In the final model (M3d), all factor loadings on the four state-like/occasion-specific factors were significant, positive, and strong (*λ* = 0.72–0.93). In contrast, factor loadings on the trait-like/stable factor were also significant and positive, but reached only weak to moderate levels (*λ* = 0.28–0.48; Fig. 1). Each HBI-8 item had at least adequate reliability (0.75–0.93) in all four waves (Geiser, 2021). With respect to Aim 1, true state-like scores for all items contained higher degree of state-like/occasion-specific variance (69–93%). Accordingly, the trait-like/stable factor explained lower rates of true score variances (7–31%). Overall, the highest levels of true score variance attributable to state-like/occasion-specific factors were observed at wave 1 (81–93%), wave 2 (77–91%), and wave 4 (78–91%), whereas the influence of the trait-like/stable factor, although still low, was most pronounced at wave 3 (13–31%; Table 3). These results suggest that CSB may be more likely to be a state-like/occasion-specific phenomenon than a trait-like/stable one.

**Table 3.** Reliability, consistency and occasion-specificity of each Hypersexual Behavior Inventory (HBI-8) item

Item	Wave	Reliability (*R*^2^)	Consistency		Occasion-specificity	
			*Con* (*SE*)	%	*Spe* (*SE*)	%
1. Even though I promised myself I would not repeat a sexual behavior, I find myself returning to it over and over again	1	0.83 (0.02)***	0.16 (0.06)**	19%	0.67 (0.06)***	81%
	2	0.80 (0.02)***	0.19 (0.06)**	23%	0.62 (0.07)***	77%
	3	0.75 (0.03)***	0.23 (0.07)**	31%	0.52 (0.07)***	69%
	4	0.81 (0.03)***	0.18 (0.06)**	22%	0.63 (0.07)***	78%
2. I sacrifice things I really want in life in order to be sexual	1	0.83 (0.02)***	0.16 (0.05)**	19%	0.67 (0.06)***	81%
	2	0.80 (0.03)***	0.19 (0.06)**	23%	0.61 (0.07)***	77%
	3	0.75 (0.03)***	0.23 (0.07)**	31%	0.52 (0.07)***	69%
	4	0.81 (0.03)***	0.18 (0.06)**	22%	0.63 (0.07)***	78%
3. I turn to sexual activities when I experience unpleasant feelings (e.g., frustration, sadness, anger)	1	0.86 (0.02)***	0.13 (0.05)**	15%	0.73 (0.05)***	85%
	2	0.84 (0.02)***	0.15 (0.05)**	18%	0.68 (0.06)***	82%
	3	0.79 (0.02)***	0.20 (0.06)**	25%	0.60 (0.07)***	75%
	4	0.84 (0.02)***	0.15 (0.05)**	18%	0.69 (0.07)***	82%
4. When I feel restless, I turn to sex in order to soothe myself	1	0.87 (0.02)***	0.12 (0.04)**	14%	0.76 (0.05)***	86%
	2	0.85 (0.02)***	0.14 (0.05)**	16%	0.71 (0.06)***	83%
	3	0.81 (0.02)***	0.18 (0.06)**	22%	0.62 (0.07)***	77%
	4	0.85 (0.02)***	0.14 (0.05)**	16%	0.72 (0.06)***	84%
5. My sexual thoughts and fantasies distract me from accomplishing important tasks	1	0.91 (0.02)***	0.08 (0.03)*	9%	0.83 (0.04)***	91%
	2	0.89 (0.02)***	0.10 (0.04)*	11%	0.79 (0.06)***	89%
	3	0.86 (0.03)***	0.14 (0.05)**	16%	0.72 (0.07)***	84%
	4	0.90 (0.02)***	0.10 (0.04)*	11%	0.80 (0.05)***	89%
6. Even though my sexual behavior is irresponsible or reckless, I find it difficult to stop	1	0.90 (0.02)***	0.09 (0.04)*	10%	0.82 (0.05)***	90%
	2	0.88 (0.02)***	0.11 (0.04)*	12%	0.78 (0.06)***	88%
	3	0.85 (0.02)***	0.14 (0.05)**	17%	0.70 (0.07)***	83%
	4	0.89 (0.02)***	0.11 (0.04)*	12%	0.78 (0.06)***	88%
7. My sexual cravings and desires feel stronger than my self-discipline	1	0.92 (0.01)***	0.07 (0.03)*	8%	0.85 (0.04)***	92%
	2	0.91 (0.02)***	0.09 (0.04)*	10%	0.82 (0.05)***	90%
	3	0.87 (0.02)***	0.12 (0.05)*	14%	0.75 (0.07)***	86%
	4	0.91 (0.02)***	0.08 (0.04)*	9%	0.83 (0.05)***	91%
8. I use sex as a way to try to help myself deal with my problems	1	0.93 (0.02)***	0.07 (0.03)*	7%	0.86 (0.04)***	93%
	2	0.91 (0.02)***	0.08 (0.03)*	9%	0.83 (0.04)***	91%
	3	0.88 (0.02)***	0.11 (0.04)**	13%	0.77 (0.06)***	87%
	4	0.91 (0.02)***	0.08 (0.03)*	9%	0.83 (0.05)***	91%

*Notes: Con* (*SE*): standardized consistency estimate (standard error). *Spe* (*SE*): standardized occasion-specificity estimate (standard error). Standardized consistency and occasion-specificity estimates might not add up to the reliability estimate due to rounding. Percentage values (%) indicate the proportion of variance in true state scores due to consistency (i.e., stable, and time-invariant trait-like effects) and occasion-specificity (i.e., time-varying state-like effects). Level of significance: *p < 0.050; **p < 0.010; ***p < 0.001.

As part of addressing Aim 2, significant and positive autoregressive effects were shown in M3d, with strong effects between waves 1–2 (*β* = 0.55) and waves 2–3 (*β* = 0.65), and with moderate strength between waves 3–4 (*β* = 0.36; Fig. 1). It is also important to consider how the inclusion of autoregressive effects between consecutive wave-specific state-like/occasion-specific residual factors affected the model structure, compared with the LST model (M3a) without such effects (see Supplementary Table 6). In the M3a model, significant, positive, moderate-to-strong factor loadings emerged for both the trait-like/stable CSB factor (*λ* = 0.43–0.80) and the wave-specific state-like/occasion-specific residual factors (*λ* = 0.41–0.87). The strongest trait-like/stable loadings occurred at waves 2 and 3 (*λ* = 0.71–0.80), whereas the highest state-like/occasion-specific loadings appeared at waves 1 and 4 (*λ* = 0.67–0.87). Accordingly, the trait-like/stable factor explained more true-score variance at waves 2 and 3 (59–79%), while variance attributable to state-like/occasion-specific factors was the highest at wave 1 (51–66%) and, most prominently, at wave 4 (68–80%).

### Sensitivity analyses

Sensitivity analyses were warranted to assess the impact of excluding a substantial number of individuals due to missing values on HBI-8 items at wave 3 (*N* = 808–813). Significant differences in sociodemographic factors and HBI-8 total scores were observed between the final sample and those excluded for missing HBI-8 values (Supplementary Tables 7 and 8). For example, the proportions of females and individuals in cohabiting romantic relationships were lower, whereas the proportion of individuals with tertiary or higher education was higher in the final sample (Supplementary Table 7). In addition, HBI-8 total scores were significantly but only weakly to moderately higher in the final sample at wave 2 (*d* = 0.49) and wave 3 (*d* = 0.28) compared to individuals excluded due to missing HBI-8 data. In contrast, at wave 1 this excluded group showed significantly but weakly (*d* = 0.20) higher HBI-8 scores than the final sample (Supplementary Table 8).

Due to these differences, we conducted additional sensitivity analyses and repeated the primary analyses, disregarding the inclusion criterion of valid HBI-8 responses and using HBI-8 scores from waves 1, 2, and 4 only. Similar patterns of bivariate correlations between HBI-8 total scores emerged as in the main analyses (Supplementary Table 9). A significant, positive, and moderate correlation was found between waves 1 and 2 (*r* = 0.31). Wave 4 scores showed positive but only weak correlations with the scores from the first two waves (*r* = 0.03–0.07), and this association reached significance only for the correlation with wave 2. Similar to the main analyses, sensitivity latent growth analysis also indicated a significant but small (*d* = 0.05–0.18) decline in CSB from waves 1 to 4 (Supplementary Table 10, Supplementary Fig. 5). However, a different growth curve emerged compared to the main analyses: the most pronounced, yet still small, decrease occurred between waves 1 and 2, with only negligible change between waves 2 and 4.

In line with the main analyses, residual invariance (*χ2* (309) = 709.379, *p* < 0.001; *CFI* = 0.992; *TLI* = 0.993; *RMSEA* [90% *CI*] = 0.027 [0.024; 0.029]; *SRMR* = 0.060) was also achieved in the sensitivity analyses for the one-factor model (Supplementary Table 11). Consistent with the main analyses, the LST model with one-trait factor and autoregressive effects (*χ2* (309) = 707.822, *p* < 0.001; *CFI* = 0.992; *TLI* = 0.993; *RMSEA* [90% *CI*] = 0.027 [0.024; 0.029]; *SRMR* = 0.062) was also the best-fitting model in the sensitivity analyses (Supplementary Table 11). This model demonstrated adequate to optimal fit. In this model, each HBI-8 item had at least adequate reliability (0.72–0.92), with strong, positive, and significant loadings on state residual factors (*λ* = 0.84–0.95) and weak, non-significant loadings on the trait factor (*λ* = 0.08–0.14). Accordingly, true score variances were largely (97–99%) explained by state-like/occasion-specific factors. Consistent with the main analyses, a significant and positive autoregressive effect emerged between wave 1 and wave 2, with a moderate effect size (*β* = 0.47) that was comparable to that observed in the main analyses. The association between wave 2 and wave 4 was also significant and positive, albeit weak (*β* = 0.22; Supplementary Table 12). Overall, the state-like conceptualization of CSB was also supported in the sensitivity analyses, and the strong factor loadings associated with the state-like/occasion-specific residual factors were reproduced similarly to the main analyses. However, the trait-like/stable effects were substantially weaker in the sensitivity analyses compared with those observed in the main analyses.

## Discussion

### Contribution of the present study

This study examined the extent to which CSB is best characterized as a trait-like/stable or state-like/occasion-specific construct, enhancing the understanding of its longitudinal course. Few studies have investigated CSB longitudinally (see Supplementary Table 2), fewer yet in representative samples (Grubbs, Kraus, Perry, Lewczuk, & Gola, 2020; Thompson et al., 2015), and most have been limited to evaluations of correlations (Ballester-Arnal et al., 2013; Reid et al., 2012; Rosansky et al., 2022) and change patterns between repeated measurements (Koós et al., 2022; Kürbitz et al., 2022; Smith et al., 2014; Thompson et al., 2015). Moreover, only a small number of studies have examined periods extending beyond one year (Gomez et al., 2024; Thompson et al., 2015). Thus, the present study extends understanding by examining CSB in a representative sample of young adults in a hybrid model over a three-year period.

While only modest temporal stability was indicated based on correlations between consecutive waves (with the highest approaching the lower end of the strong range), the magnitude of wave-to-wave changes ranged between negligible and small. This apparent discrepancy suggested that the observed temporal variability could arise from multiple sources. The LST framework enabled us to clarify this ambiguity by distinguishing trait-like stability from state-like/occasion-specific fluctuations and measurement error. This approach provides a more precise account of the longitudinal course of CSB severity across measurement occasions.

The longitudinal structure of the CSB was best described by a model that included four state-like/occasion-specific CSB factors linked with autoregressive effects in addition to a general, non-symptom-specific trait-like/stable factor. The variance in true CSB symptom scores was primarily attributable to state-like/occasion-specific effects. This suggests that CSB is more likely to be a state-like/occasion-specific construct among young adults from the general population. Among them, situational influences (e.g., negative life events, changes in relationship status, fluctuations in affective states and sexual desire, access to sexualized online content) may contribute to the longitudinal course of CSB (Geiser et al., 2021; Grant Weinandy et al., 2023; Mestre-Bach & Potenza, 2023; Wizła & Lewczuk, 2024). The role of state-like/occasion-specific influences in the longitudinal course of CSB was further indicated by the finding that, without autoregressive effects in the model, trait-like/stable influences accounted for a larger proportion of variance, particularly at waves 2 and 3. This pattern suggests that the contribution of the temporally unchanging, habitual CSB level captured by the trait-like/stable factor decreases once short-term situational stability or the carry-over effect arising from the preceding CSB state is considered.

The predominance of state-like/occasion-specific effects is consistent with previous findings that reported a longitudinally fluctuating and transient course of CSB (e.g., an increase in symptom severity was subsequently followed by a decrease, limited diagnostic consistency over time) (Castro-Calvo et al., 2023; Kürbitz et al., 2022). Moreover, the greater contribution of state-like/occasion-specific factors to longitudinal symptom variability may help contextualize the significant and small decrease in CSB severity from the first to the last measurement. Comparable significant decreases have been documented in previous longitudinal investigations of CSB (Castro-Calvo et al., 2023; Deng et al., 2021; Grassi et al., 2024; Konkolÿ Thege et al., 2015; Smith et al., 2014; Thompson et al., 2015). It may also indirectly explain why natural recovery may occur and why a relatively low prevalence of persistently and chronically severe CSB in adult community samples has been reported (Castro-Calvo et al., 2023; Konkolÿ Thege et al., 2015). Overall, these patterns suggest that symptom escalation may be temporary for many young adults in community samples and largely shaped by situational and contextual factors (e.g., negative life events and affective states, changes in relationship status) (Grant Weinandy et al., 2023; Mestre-Bach & Potenza, 2023; Wizła & Lewczuk, 2024). Accordingly, spontaneous remission may occur as these influences change or diminish, without the need for formal medical or psychological intervention in some cases (Castro-Calvo et al., 2023; Konkolÿ Thege et al., 2015).

### Theoretical and conceptual implications

The state-like nature of CSB may have important theoretical and conceptual implications. Specifically, CSB may share similarities with potentially addictive behaviors and impulse control disorders. Prior studies of potentially addictive behaviors in general population samples have demonstrated pronounced longitudinal transiency (Konkolÿ Thege et al., 2015), while other investigations (e.g., using BLS data) have suggested that a substantial proportion of longitudinal symptom variance is attributable to state-like/occasion-specific factors (Horváth et al., 2024). Consistent with this, impulse control disorders as defined in the ICD-11 (e.g., pyromania, kleptomania, intermittent explosive disorder) involve symptom features that highlight the relevance of situational and affective states preceding or accompanying problematic behaviors (e.g., heightened affective arousal and tension, unplanned aggressive behavior in response to perceived social obstacles) (World Health Organization, 2019). Findings from intensive longitudinal studies (e.g., experience sampling method, ecological momentary assessment) similarly indicate that impulsive behaviors and addictive features (e.g., craving) can fluctuate rapidly within individuals and covary with affective (e.g., positive and negative emotions), cognitive (e.g., risk preferences, attentional biases, motives), and situational factors (Alvarez, Hafezi, Bonagura, Kleiman, & Konova, 2022; Hawker, Dias, Merkouris, Rodda, & Dowling, 2025; Lo, Chan, & Cho, 2026; Zhou, Zhou, Zhou, Shen, & Zhang, 2022). Taken together, these patterns are consistent with cue-reactivity models of impulsive and addictive behaviors (Carter & Tiffany, 1999; Jansen, 1998). The similarity of CSB to other impulse control disorders and addictive disorders at the symptomatic and behavioral level (e.g., impaired control over behavior, significant intra- and interpersonal impairment) may, at least in part, reflect shared neurobiological characteristics. In this regard, both impulsive and addictive disorders have been associated with alterations in dopaminergic and serotonergic systems, dysregulation of hypothalamic-pituitary-adrenal (HPA) axis functioning, and overlapping neural circuits related to cue reactivity, craving, and cue-related reward processing (e.g., ventral striatum, amygdala) (Brewer & Potenza, 2008; Kraus, Voon, & Potenza, 2016).

The inclusion of potentially situationally contingent and event-specific items in the HBI-8 (e.g., sexual behavior triggered by unpleasant emotions such as frustration, sadness, or anger) may also have been sensitive to identifying sporadic and temporary symptom expressions (Bőthe et al., 2019; Reid et al., 2011). Similarly, the ICD-11 recognizes, beyond diagnostic criteria, the relevance of emotional and behavioral cues in CSBD-related sexual behavior (e.g., depressive and anxious states, boredom, loneliness) (World Health Organization, 2019). Affective responses following sexual behavior (e.g., sexual regret) may also warrant consideration, as they may contribute to symptom perception.

At the same time, the pronounced state-like nature of CSB may be less compatible with the repetitive and persistent symptom patterns typically described for obsessive–compulsive disorders in the ICD-11 (World Health Organization, 2019). However, cautious interpretation is warranted, as the measurement of CSB in the present study might have been less capable of capturing more compulsive functioning that can be present in severe stages of CSB (e.g., tolerance) (Bőthe et al., 2022; Sassover & Weinstein, 2020).

### Contextual and study-specific characteristics

Multiple contextual and study-specific factors should be considered when interpreting and generalizing the present findings. In the representative sample of the present study, 66.35, 71.95, 70.27, and 82.59% of valid respondents did not indicate the presence of any CSB symptoms in waves 1–4, respectively. It is plausible that the lower severity of CSB might account for the superior effect of state-like/occasion-specific factors (Castro-Calvo et al., 2023). In a clinical sample with higher CSB severity, CSB may be a more trait-like/stable construct, warranting further studies. In contrast, it is also conceivable that the applied inclusion criterion requiring continuous sexual activity across all four waves may have reduced state-like/occasion-specific influences on symptom fluctuations. Specifically, greater variability in CSB symptoms (and therefore a stronger contribution of state-like/occasion-specific factors) might have been observed if participants with periods of enforced abstinence had been included. Accordingly, individuals with intermittent sexual activity or episodes of enforced abstinence may display different fluctuation patterns in CSB symptoms, indicating that the balance of trait-like/stable and state-like/occasion-specific effects on CSB trajectories may differ from what was observed in the present sample.

Moreover, the state-like/occasion-specific effects of CSB may relate to the young-adult sample. Significant fluctuations in CSB severity may occur due to changes in sexual life (e.g., hormonal changes) and psychological and social characteristics (e.g., decreased impulsivity) longitudinally (Castro-Calvo et al., 2023). Finally, waves 2–3 of the study overlapped with the onset of the COVID-19 pandemic, which may have influenced sexual behaviors and motivations (e.g., greater role of pornography use) (Bőthe et al., 2022; Koós et al., 2022). This could relate to the moderate and strong autoregressive effects observed from the second and third state-like/occasion-specific factors, reflecting the stability of situational influences during this period. Situation-specific effects related to the COVID-19 pandemic might have led to decreases in CSB severity in subsequent measurements.

### Limitations

Methodological limitations warrant consideration. Results cannot be generalized cross-culturally, as culture-based CSB-related differences exist (Bőthe et al., 2023). Cultural norms and personal value systems surrounding sexual behavior (e.g., stricter moral or social regulations) may influence how CSB is perceived and reported, potentially leading to substantial variation across countries (Bőthe et al., 2023).

Furthermore, while the sensitivity analysis corroborated the state-like/occasion-specific nature of CSB, the high number of participants excluded from the final sample, as well as the significant differences between included and excluded individuals (e.g., gender, cohabiting romantic relationship), may limit the generalizability of the findings. In addition to attrition-related exclusions, individuals who were omitted due to missing HBI-8 data or intermittent sexual activity (e.g., periods of enforced abstinence) may have exhibited different patterns of trait-like/stable versus state-like/occasion-specific effects. Moreover, as the present findings were derived from individuals with relatively low levels of CSB, it cannot be ruled out that higher or clinically significant CSB levels would be associated with different proportions of trait-like/stable and state-like/occasion-specific variability. As such, the present results should be interpreted with caution when considering populations whose behavioural or contextual patterns may differ from those represented in the analytic sample.

Given the self-report measurement, responses may have been biased by social desirability, recall bias, sexuality-related stigma, or limited self-reflection. These factors may have contributed to lower reported CSB severity in the present study. The present study investigated CSB over a longer interval (i.e., 12 months between waves) compared to the temporal criterion of CSBD in the ICD-11 (i.e., at least six months). Finally, ICD-11 CSBD criteria were not assessed comprehensively (e.g., lack of items assessing continued engagement despite reduced satisfaction). Accordingly, there was only limited correspondence between the present CSB measurement and the ICD-11-based conceptualization of CSBD, and a comprehensive diagnostic evaluation was not performed.

### Future directions

The extent to which CSB and CSBD can be considered trait-like/stable or state-like/occasion-specific constructs among treatment-seeking individuals with higher symptom severity or in a representative sample of the entire adult population should be explored. Thus, future studies including individuals with a broader range of CSB severity are needed to examine the robustness and generalizability of the observed patterns. Future research may also examine samples with more diverse background characteristics (e.g., a broader age range, gender and sexual orientation minorities) to better understand CSB symptoms longitudinally. The use of more intensive longitudinal designs (e.g., experience sampling method, ecological momentary assessment) may also contribute to a more precise evaluation of the longitudinal course of CSB (Borgogna et al., 2025). Moreover, the application of the ICD-11-based CSBD-specific diagnostic interviews or screening instruments (e.g., the Compulsive Sexual Behavior Disorder Scale) are needed (Bőthe et al., 2020). This would facilitate a more comprehensive diagnostic evaluation. Additionally, it may be worth assessing acute situational influences (e.g., negative affective states, religious or moral judgments, and contextual influences related to increased access to sexual materials and sexual impulses, as described in the ICD-11) as well as exploring previous symptomatic severities when measuring CSBD/CSB in cross-sectional and longitudinal research. Further important goals of future research could be to examine whether the relative contributions of trait-like/stable vs. state-like/occasion-specific CSB factors to longitudinal symptom variance differ across groups with different background characteristics (e.g., gender, age, sexual-orientation) as well as across groups characterized by various sexual behaviors (e.g., partnered sex vs. solitary sexual behaviors) and sexual activity patterns (e.g., individuals with intermittent vs. continuous activity).

## Conclusions

CSB may be best considered as an essentially state-like/occasion-specific construct among a representative sample of young adults in the general population. The role of state-like/occasion-specific effects on CSB severity may have important implications for the diagnostic and screening guidelines as well as treatments related to CSBD. The state-like nature of symptoms may influence the distinction between ‘diagnostic orphans’ and individuals diagnosed with CSBD. Namely, the ICD-11 indicates that for a diagnosis of CSBD, symptoms should persist for at least six months (World Health Organization, 2019). Given the fluctuating nature of CSB, individuals experiencing significant impairment and distress for a shorter duration may not be diagnosed with CSBD. Consequently, the state-like characteristics of CSB may lead to higher false negative rates during screening (Kessler et al., 2005). Thus, further consideration of the specified timeframe for CSBD assessment in the ICD-11 might be warranted. The rather state-like nature of CSB may have important implications for the effectiveness of CSBD interventions. Although limited evidence has been available on the effectiveness of interventions (Antons et al., 2022; López-Pinar, Esparza-Reig, & Bőthe, 2025; Turner et al., 2022), the more pronounced natural fluctuation in CSB may result in a higher placebo effect (Huneke et al., 2024). This, in turn, could make it more difficult to detect symptomatic improvements between intervention groups receiving active versus control treatments, warranting future investigation.

## Supplementary material
