# Supplementary material for: Compulsive sexual behavior may be more state-like than trait-like: Findings from a three-year longitudinal representative survey
Source: J Behav Addict. 2026 Apr 15;15(2):622–35. doi: 10.1556/2006.2025.00419 (PMC13371728; doi:10.1556/2006.2025.00419)
Supplement: Supplementary file 1 [file jba-15-622-s001.pdf]

Zsolt Horváth, Z., Bőthe, B. et al.: Compulsive sexual behavior may be more state-like than trait-like: Findings from a three-year longitudinal representative survey

https://doi.org/10.1556/2006.2025.00419

Supplementary materials

Table of contents

Supplementary Fig. 1. PRISMA flow diagram for the supplementary systematic review 2

Supplementary Table 1. Methodological details of the supplementary systematic review 3

Supplementary Table 2. Characteristics of studies included in the supplementary systematic review ... 5

Supplementary Fig. 2. Detailed research procedure and process of obtaining the final sample............. 7

Supplementary Table 3. Detailed description of the measurement invariance models of the Hypersexual Behavior Inventory (HBI-8) 8

Supplementary Fig. 3. Graphical illustration of the tested latent state-trait (LST) models. 11

Supplementary Fig. 4. Estimated means of the total score of the of the short version of the Hypersexual Behavior Inventory (HBI-8) based on latent growth modelling 13

Supplementary Table 4. Model fit indices of the longitudinal invariance and latent state-trait (LST) models... 14

Supplementary Table 5. Factor loadings, correlations, and internal reliability of the one-factor model of the short version of the Hypersexual Behavior Inventory (HBI-8).. 16

Supplementary Table 6. Factor loadings, reliability, consistency and occasion-specificity of each Hypersexual Behavior Inventory (HBI-8) item in the LST model with one trait factor, without method factors and autoregressive effects (M3a model). 17

Supplementary Table 7. Sensitivity analysis: comparison of individuals included in and excluded from the final sample on socio-demographics in wave 1 19

Supplementary Table 8. Sensitivity analyses: comparisons in terms of the total score of the eight-item Hypersexual Behavior Inventory (HBI-8) 20

Supplementary Table 9. Sensitivity analysis: descriptive statistics and pairwise correlations between the eight-item Hypersexual Behavior Inventory (HBI-8) scores in Waves 1, 2 and 4 (W1-W4)................ 21

Supplementary Table 10. Sensitivity analysis: latent growth modelling based on the total score of the Hypersexual Behavior Inventory (HBI-8) across waves 1, 2 and 4.. 22

Supplementary Fig. 5. Estimated means of the total score of the of the short version of the Hypersexual Behavior Inventory (HBI-8) based on latent growth modelling across waves 1, 2 and 4...................... 23

Supplementary Table 11. Sensitivity analysis: model fit indices of the longitudinal invariance models related to the one-factor model and the latent state-trait (LST) model without method factors and with one trait factor. 24

Supplementary Table 12. Sensitivity analysis: factor loadings, reliability, consistency and occasion-specificity of each Hypersexual Behavior Inventory (HBI-8) item 25

References.. 27

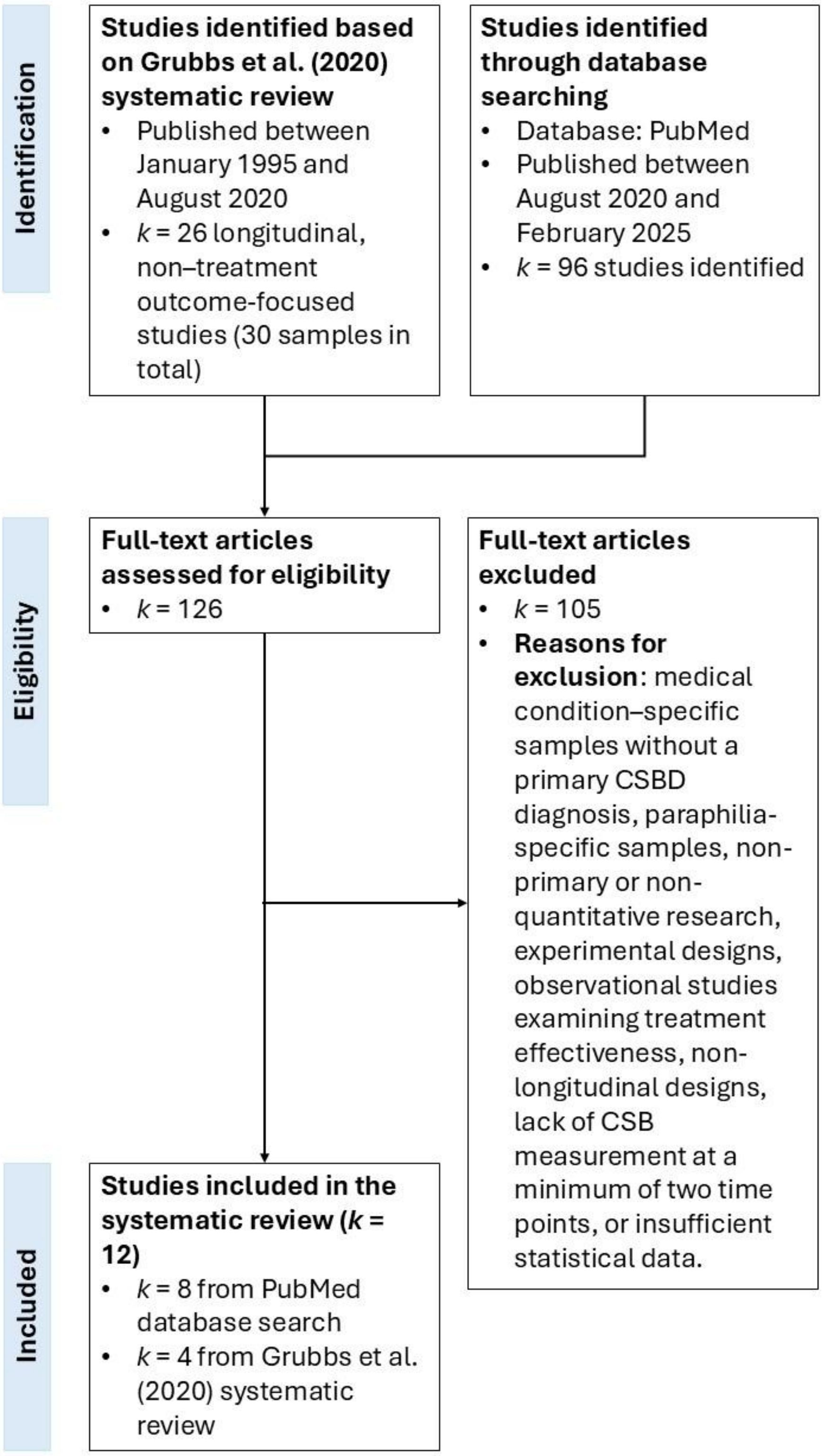

Supplementary Fig. 1. PRISMA flow diagram for the supplementary systematic review

Supplementary Table 1. Methodological details of the supplementary systematic review

**Aim**	To identify and review studies investigating the longitudinal course of compulsive sexual behavior (CSB).
**Sources of identified studies**	Prior to the present study, Grubbs and colleagues conducted the most recent systematic review in 2020, which partially addressed the above-mentioned review aim. That review included literature published between January 1995 and August 2020 and identified 26 individual longitudinal studies (comprising a total of 30 separate samples) on CSB, not considering studies that focused on treatment outcomes. As part of the present supplementary review, an additional literature search was performed in PubMed to identify further relevant studies published since August 2020.
**Date of the additional literature search in PubMed**	February 26, 2025
**Search terms used in PubMed**	("hypersex*"[Title/Abstract] OR "sex addict*"[Title/Abstract] OR "sexu* addict*"[Title/Abstract] OR "sexu* compul*"[Title/Abstract] OR "compul* sex"[Title/Abstract] OR "compul* sexu*"[Title/Abstract] OR "sex impul*"[Title/Abstract] OR "sexu* impul*"[Title/Abstract] OR "impul* sex"[Title/Abstract] OR "impul* sexu*"[Title/Abstract] OR "uncon* sex"[Title/Abstract] OR "uncon* sexu*"[Title/Abstract] OR "excessive sex"[Title/Abstract] OR "excessive sexu*"[Title/Abstract] OR "sexu* preoccupation"[Title/Abstract] OR "out of control sexu*"[Title/Abstract]) AND ("longitudinal"[Title/Abstract] OR "prospective"[Title/Abstract] OR "retrospective"[Title/Abstract] OR "long term"[Title/Abstract] OR "cross lagged"[Title/Abstract] OR "growth"[Title/Abstract] OR "trajector*"[Title/Abstract] OR "cohort"[Title/Abstract] OR "case control"[Title/Abstract] OR "follow up"[Title/Abstract]) AND 2020/08/01:3000/12/31[Date - Publication].
**Inclusion criteria for eligible studies**	1. Studies had to include community samples, participants with a primary diagnosis of compulsive sexual behavior disorder (CSBD), or participants with a psychiatric diagnosis potentially comorbid with CSBD (e.g., addictive disorders, impulse control disorders, personality disorders).<br>2. Studies had to be original, primary research employing a quantitative methodology.<br>3. Studies had to utilize an observational, longitudinal design.<br>4. Studies had to assess CSB (not necessarily the clinical diagnosis of CSBD) with a standardized instrument at a minimum of two consecutive time points.
**Exclusion criteria**	1. Studies that included individuals with neurological, somatic, or other medical conditions that may be associated with the development of CSB (e.g., Parkinson's disease), or that are not directly associated with CSB (e.g., AIDS-specific samples), as well as samples including individuals with paraphilias (e.g., child sex offenders).<br>2. Studies that constituted non-primary or non-quantitative research (e.g., reviews, meta-analyses, case studies).<br>3. Studies that employed experimental designs (e.g., treatment or intervention studies, laboratory experiments) or observational designs that focused on treatment effectiveness or treatment-related change.<br>4. Studies that did not employ a longitudinal design (e.g., cross-sectional studies); did not assess CSB at a minimum of two time points within a longitudinal framework.<br>5. Studies that focused exclusively on a specific form of sexual activity or a specific behavioral subtype (e.g., problematic pornography use).

	6. Studes that did not report sufficient statistical information (e.g., means and standard deviations, proportions, or correlations) to evaluate stability or change in CSB.
**Assessment of eligibility**	Two authors (ZH, SAD) independently reviewed the articles to assess their eligibility based on the predefined inclusion and exclusion criteria. Inter-rater agreement was reached for 107 studies (87.71%), indicating a moderate level of inter-rater reliability (Cohen's $\kappa = 0.48$). For the 15 studies (12.30%) where the reviewers initially disagreed, a consensus decision was achieved through discussion with a third author (BB).
**Information extracted from relevant studies**	• Authors and title<br>• Publication year<br>• Number of measurement points<br>• Interval between the first and last measurement points (in months)<br>• Sample type according to clinical severity (clinical vs. non-clinical sample)<br>• Sample type according to representativeness (representative vs. non-representative sample)<br>• Sample size<br>• Mean or median age<br>• Sex/gender<br>• CSB measurement<br>• Statistical method applied to examine the longitudinal course of CSB<br>• Qualitative interpretation of the longitudinal course of CSB

Supplementary Table 2. Characteristics of studies included in the supplementary systematic review

Study	Measure-ment points	Measure-ment interval (first–last, months)	Sample clinical status	Sample represen-tativeness	Sample size	Mean (*M*) or median (*Md*) age	Sex or gender	Compulsive sexual behavior (CSB) measurement	Statistical method	Interpretation of longitudinal CSB course
Ballester-Arnal et al., 2013	2	0.25	Non-clinical	Non-representative	91	N/A	N/A	Sexual Compulsivity Scale (SCS)	Correlation	Stability or negligible change (i.e., a strong correlation between the two measurement points)
Bőthe et al., 2021	2	6	Non-clinical	Non-representative	2 × 267	Males: *M*=29.9 Females: *M*=27.7	Mixed-sex couples: 50% female and 50% male	Hypersexual Disorder Screening Inventory (HDSI)	Correlation, mean difference	Stability or negligible change (i.e., moderate-to-strong correlations and only negligible decreases in both sexes between the two measurement points)
Deng et al., 2021	2	4	Non-clinical	Non-representative	2998	*M*=20.5	Men: 24% Women: 76%	Sexual Compulsivity Scale (SCS)	Correlation, mean difference	Significant decrease (i.e., a strong decrease and a very low correlation between the two measurement points)
Efrati, 2024	2	6	Non-clinical	Non-representative	300	*M*=15.9	Men: 39% Women: 61%	Individual-based Compulsive Sexual Behavior (I-CSB) Scale	Correlation	Stability or negligible change (i.e., a strong correlation between the two measurement points)
Gomez et al., 2024	3	24	Non-clinical	Non-representative	276	*M*=31.9	Men: 71% Women: 33% Unspecified gender: 2%	Bergen–Yale Sexual Addiction Scale (BYSAS)	Correlation	Stability or negligible change (i.e., strong correlations across measurement points)
Grassi et al., 2024	2	12	Clinical	Non-representative	37	*Md*=28	Men: 24% Women: 76%	Sex Addiction Screening Test – Revised (SAST-R)	Mean difference	Significant decrease (i.e., with a small decrase between the two measurement points)
Koós et al., 2022	3	10	Non-clinical	Non-representative	417	*M*=43.0	Men: 41% Women: 56%	Compulsive Sexual Behavior	Latent growth curve modelling	Stability or negligible change (i.e., a negligible increase indicated by the significant yet

Study	Measure-ment points	Measure-ment interval (first–last, months)	Sample clinical status	Sample represen-tativeness	Sample size	Mean (*M*) or median (*Md*) age	Sex or gender	Compulsive sexual behavior (CSB) measurement	Statistical method	Interpretation of longitudinal CSB course
							Other gender: 1%	Disorder Scale (CSBD-19)		small variance estimate and by the significant mean estimates associated with the slope effect)
Kürbitz et al., 2022	5	8	Non-clinical	Non-representative	77	*M*=32.5	Men: 40% Women: 60%	Yale-Brown Obsessive-Compulsive Scale (Y-BOCS)	Linear mixed modelling	Stability or negligible change (i.e., significant increases were observed between the first two measurement points for both genders, whereas CSB levels subsequently returned to their initial level and changed only negligibly for both genders)
Reid et al., 2012	2	0.5	Clinical	Non-representative	32	N/A	N/A	Hypersexual Disorder Diagnostic Clinical Interview (HD-DCI)	Correlation	Stability or negligible change (i.e., a strong correlation between the two measurement points)
Rosansky et al., 2022	2	6	Non-clinical	Non-representative	337	*M*=37.2	Men: N/A Women: 76%	Hypersexual Behavior Inventory (HBI)	Correlation	Stability or negligible change (i.e., a strong correlation between the two measurement points)
Smith et al., 2014	3	6	Non-clinical	Non-representative	258	N/A	Men: 100%	Minnesota Impulsive Disorder Inventory (MIDI)	Generalized estimating equations (GEE)	Significant decrease (i.e., no effect size was reported)
Thompson et al., 2015	2	36	Non-clinical	Represen-tative	572	*M*=18.6	Men: 100%	Sexual Compulsivity Scale (SCS)	Repeated measures analysis of variance (ANOVA)	Significant decrease (i.e., no effect size was reported)

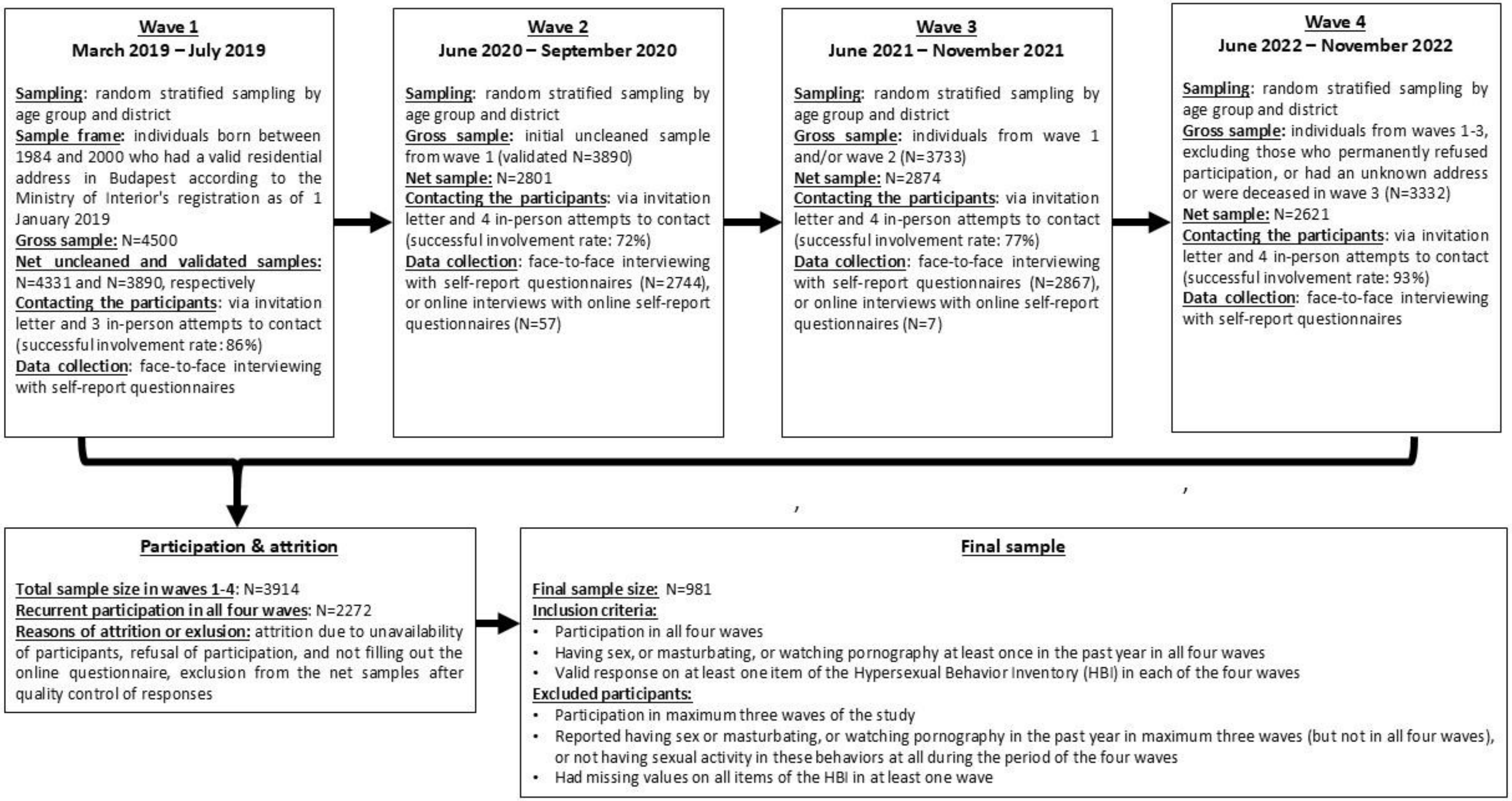

Supplementary Fig. 2. Detailed research procedure and process of obtaining the final sample

Supplementary Table 3. Detailed description of the measurement invariance models of the Hypersexual Behavior Inventory (HBI-8)

	One-factor model (M1)	Three-factor model (M2)
**Latent factor(s) of the model**	A general factor of compulsive sexual behavior (CSB).	The three correlated factors of consequences, control, and coping.
**Observed indicators of the model**	The indicator variables of the latent factor were the items of HBI-8, defined as ordinal categorical variables. That is, all 8 items of the HBI-8 loaded on the general factor of CSB. Since for some variables the highest frequency response category (5=Very often) did not contain any valid responses (N=0) in some cases, all observed indicator variables were recoded in a way that they included four response categories (1=Never, 2=Rarely, 3=Sometimes, 4=Often or Very often)	The indicator variables of the latent factor were the items of HBI-8, defined as ordinal categorical variables. The consequences factor was defined by two items, while three-three items loaded on the factors of control and coping. Since for some variables the highest frequency response category (5=Very often) did not contain any valid responses (N=0) in some cases, all observed indicator variables were recoded in a way that they included four response categories (1=Never, 2=Rarely, 3=Sometimes, 4=Often or Very often)
**Literature background of the model**	According to the literature, total scores are calculated for both the full 19-item HBI and the abbreviated HBI-8, which is in line with the one-factor structure (Grubbs et al., 2023; Reid et al., 2011).	Several previous studies supported and confirmed a three-factor structure of the full 19-item HBI (Ballester-Arnal et al., 2019; Bőthe et al., 2019; Reid et al., 2011).
**Estimation method**	Due to the ordinal and skewed distribution of the indicator variables, the models were estimated using the weighted least squares means and variances adjusted (WLSMV) procedure.	
**Literature background of the tested invariance models**	Liu et al., 2017	
**Configural invariance model (M1e, M2e)**	The first item of the HBI-8 (i.e., the first items of the latent factors) was selected as marker items in all four waves, so their factor loadings were fixed at 1.00, while the factor loadings of the other indicator variables were estimated freely. For these marker variables, the thresholds between the first and second response category and between the second and third response category were fixed in equal across measurement occasions. For the other items, the threshold between the first and second response category was fixed in equal across measurement occasions. In the first wave, the residual variances of all observed variables were fixed at 1.00, while in the second, third and fourth waves, the residual variances	The first items of the latent factors (i.e., items 1-3 of the HBI-8) were selected as marker items in all four waves, so their factor loadings were fixed at 1.00, while the factor loadings of the other indicator variables were estimated freely. For these marker variables, the thresholds between the first and second response category and between the second and third response category were fixed in equal across measurement occasions. For the other items, the threshold between the first and second response category was fixed in equal across measurement occasions. In the first wave, the residual variances of all observed variables were fixed at 1.00, while in the second, third and fourth waves, the residual variances

	One-factor model (M1)	Three-factor model (M2)
	of the observed indicators defining the latent factors were estimated freely.	of the observed indicators defining the latent factors were estimated freely.
**Metric invariance model (M1f, M2f)**	The factor loadings of each item were fixed in equal over time, and the first item remained as a marker item, so its factor loading was fixed at 1.00. For the marker variable, the thresholds between the first and second response category and between the second and third response category were fixed in equal across measurement occasions. For the other items, the threshold between the first and second response category was fixed in equal across measurement occasions. In the first wave, the residual variances of all variables were fixed at 1.00, while in the second, third and fourth waves, the residual variances of the observed indicators were estimated freely.	The factor loadings of each item were fixed in equal over time, and the first items of each latent factor remained as marker items, so their factor loading was fixed at 1.00. For the marker variables, the thresholds between the first and second response category and between the second and third response category were fixed in equal across measurement occasions. For the other items, the threshold between the first and second response category was fixed in equal across measurement occasions. In the first wave, the residual variances of all variables were fixed at 1.00, while in the second, third and fourth waves, the residual variances of the observed indicators were estimated freely.
**Scalar invariance model (M1g, M2g)**	The factor loadings of each item were fixed in equal over time, and the first item remained as a marker item, so its factor loading was fixed at 1.00. For each item, the thresholds between response categories were fixed in equal values over time. In the first wave, the residual variances of all variables were fixed at 1.00, while in the second, third and fourth waves, the residual variances of the observed indicators were estimated freely.	The factor loadings of each item were fixed in equal over time, and the first items of each latent factor remained as marker items, so their factor loadings were fixed at 1.00. For each item, the thresholds between response categories were fixed in equal values over time. In the first wave, the residual variances of all variables were fixed at 1.00, while in the second, third and fourth waves, the residual variances of the observed indicators were estimated freely.
**Residual invariance model (M1h, M2h)**	The factor loadings of each item were fixed in equal over time, and the first item remained as a marker item, so its factor loading was fixed at 1.00. For each item, the thresholds between response categories were fixed in equal values over time. Residual variances of all observed variables were fixed at 1.00.	The factor loadings of each item were fixed in equal over time, and the first items of each factor remained as marker items, so their factor loading were fixed at 1.00. For each item, the thresholds between response categories were fixed in equal values over time. Residual variances of all observed variables were fixed at 1.00.
**Further notes for the invariance models (latent means, correlations)**	For each of the invariance models, the mean of the latent factor measured in the first wave were fixed at 0, while in the second, third and fourth waves the means of the latent factors were freely estimated. The three latent factors were freely correlated with each other over time.<br>The only difference from the invariance models proposed by Liu et al. was that we did not allow for unique residual correlations of each item with itself over time in any of the invariance models. The reason for this was that the assumption of correlated uniqueness in	For each of the invariance models, the mean of the latent factors measured in the first wave were fixed at 0, while in the second, third and fourth waves the means of the latent factors were freely estimated. A given factor was allowed to correlate with itself over time as well as to correlate with other factors within a given measurement occasion, but it was not allowed to correlate with other factors at other measurement points. As in the one-factor first-order model, unique residual correlations of each item with itself over time were not estimated.

	One-factor model (M1)	Three-factor model (M2)
	latent state-trait (LST) models may contribute to a number of statistical and methodological problems, e.g. factors may lose their originally intended meaning or become confounded and thus cannot be considered as a true LST model, can result in having less parsimonious models, specific variance related to method effects cannot be captured, lack of correlation between method effects may not be an appropriate assumption, and lower levels of reliability of observed indicators may be presented (Geiser & Lockhart, 2012).	
**Comparisons of the measurement invariance models**	Chi-square difference test was calculated to compare model fit of consecutive invariance models by using the DIFFTEST option in Mplus (Muthén & Muthén, 2017). Moreover, the degree of change between consecutive measurement invariance models was examined in terms of the comparative fit index (CFI), standardized root mean square residual (SRMR), and root mean square error of approximation (RMSEA). The more restrictive invariance model representing the equality of more statistical parameters was accepted if the decrease in the CFI and SRMR was ≤0.010, and if the increase in the RMSEA was ≤0.015 (Chen, 2007).	

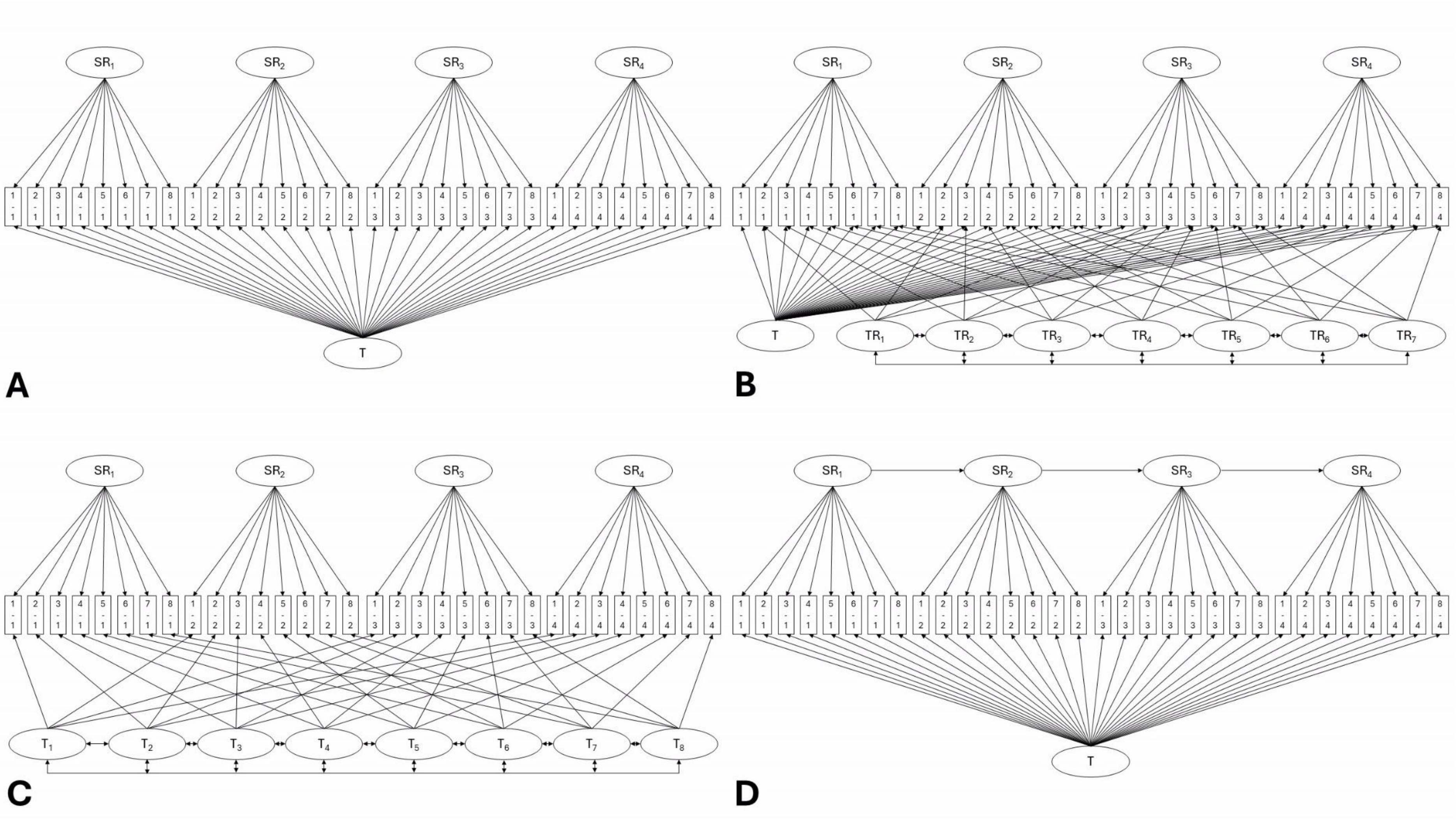

Supplementary Fig. 3. Graphical illustration of the tested latent state-trait (LST) models.

Notes. Model A: LST model with one general trait-like/stable factor and without method factors. Model B: LST model with M-1 correlated latent method factors (with one general trait-like/stable factor and seven correlated method factors). Model C: LST model with eight correlated indicator-specific trait-like/stable factors. Model D: LST model with one general trait-like/stable factor (no

method factors) and autoregressive effects. Notes. Single-headed arrows between latent factors (in ellipse) and observed variables (in square) indicate state-like/occasion-specific residual factor loadings (δ), or trait-like/stable factor loadings (λ), or method factor loadings (γ). Single-headed arrows between latent factors indicate autoregressive effects between state-like/occasion-specific residual factors (β). Double headed arrows represent correlations (r). SR_1-SR_4: latent state-like/occasion-specific residual factors. T: latent general trait-like/stable factor. T_1-T_8: latent indicator-specific trait-like/stable factors. TR_1-TR_7: latent indicator specific trait-like/stable residual factors. The upper value represents the item number in the Hypersexual Behavior Inventory (see Table 4), while the lower value indicates the measurement wave. In each model, the first item of the Hypersexual Behavior Inventory was a marker item for SR_1-SR_4, thus its factor loading (δ) was fixed at 1.00 on SR_1-SR_4. The factor loadings (δ) of the other items were fixed in equal over time. All factor loadings (λ, γ) on T, T_1-T_8, and TR_1-TR_7 were fixed at 1.00. For each item, the thresholds between response categories were fixed in equal values over time. Residual variances of all observed variables were fixed at 1.00. The latent mean of SR_1, T, T_1-T_8, and TR_1-TR_7 were fixed at 0.00, while latent means of SR_2-SR_4 were freely estimated. Models A-C were tested based on the work of Geiser and Lockhart (2012). However, the proposed LST models with correlated uniqueness and with orthogonal method factors were not tested due to statistical and methodological problems associated with these models (Geiser & Lockhart, 2012).

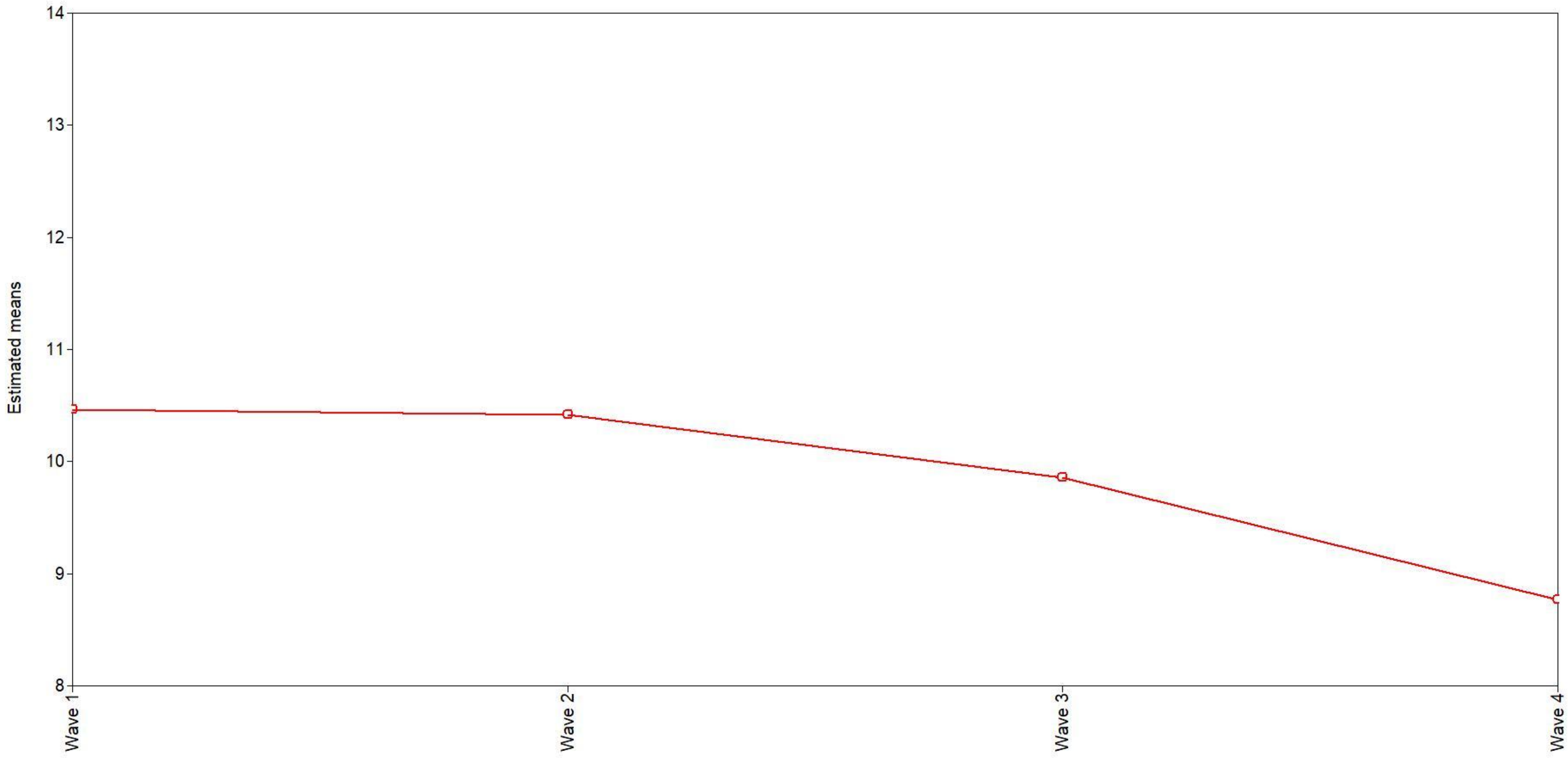

Supplementary Fig. 4. Estimated means of the total score of the of the short version of the Hypersexual Behavior Inventory (HBI-8) based on latent growth modelling

Supplementary Table 4. Model fit indices of the longitudinal invariance and latent state-trait (LST) models

	χ^2 (*df*)	*p*	*CFI*	*TLI*	*RMSEA* [90% *CI*]	*SRMR*	Comparison	$\Delta\chi^2$ (*df*)	*p*	Δ*CFI*	Δ*TLI*	Δ*RMSEA*	Δ*SRMR*
**One-factor model (M1)**													
M1a: Wave 1	73.892 (20)	< 0.001	0.997	0.996	0.061 [0.047; 0.077]	0.016	-	-	-	-	-	-	-
M1b: Wave 2	47.463 (20)	< 0.001	0.997	0.996	0.044 [0.028; 0.060]	0.018	-	-	-	-	-	-	-
M1c: Wave 3	43.791 (20)	0.002	0.997	0.995	0.041 [0.024; 0.057]	0.024	-	-	-	-	-	-	-
M1d: Wave 4	26.290 (20)	0.157	0.999	0.999	0.021 [0.000; 0.041]	0.021	-	-	-	-	-	-	-
M1e: Configural invariance	511.561 (458)	0.042	0.998	0.998	0.013 [0.003; 0.019]	0.059	-	-	-	-	-	-	-
M1f: Metric invariance	540.788 (479)	0.026	0.998	0.998	0.013 [0.005; 0.019]	0.060	M1e vs. M1f	46.918 (21)	0.001	0.000	0.000	0.000	0.000
M1g: Scalar invariance	589.345 (524)	0.025	0.998	0.998	0.013 [0.005; 0.019]	0.060	M1f vs. M1g	58.126 (45)	0.091	0.000	0.000	0.000	0.000
M1h: Residual invariance	635.755 (548)	0.006	0.997	0.997	0.015 [0.009; 0.020]	0.062	M1g vs. M1h	54.176 (24)	< 0.001	-0.001	-0.001	-0.002	-0.002
**Three-factor model (M2)**													
M2a: Wave 1[1]	52.467 (17)	< 0.001	0.998	0.997	0.054 [0.038; 0.071]	0.013	-	-	-	-	-	-	-
M2b: Wave 2[1]	45.878 (17)	< 0.001	0.997	0.995	0.049 [0.032; 0.066]	0.018	-	-	-	-	-	-	-
M2c: Wave 3[1]	41.084 (17)	0.001	0.997	0.994	0.045 [0.027; 0.062]	0.024	-	-	-	-	-	-	-
M2d: Wave 4[1]	25.095 (17)	0.093	0.999	0.999	0.026 [0.000; 0.046]	0.021	-	-	-	-	-	-	-
M2e: Configural invariance[1]	6199.777 (434)	< 0.001	0.785	0.754	0.136 [0.133; 0.139]	0.283	-	-	-	-	-	-	-
**Latent state-trait (LST) models (M3)**													
M3a: One trait factor without method factors	785.245 (553)	< 0.001	0.991	0.992	0.024 [0.020; 0.028]	0.102	-	-	-	-	-	-	-
M3b: One trait factor with seven correlated method factors[1]	738.356 (525)	< 0.001	0.992	0.992	0.024 [0.020; 0.028]	0.100	-	-	-	-	-	-	-
M3c: Eight indicator-specific trait factors[1]	731.727 (518)	< 0.001	0.992	0.992	0.024 [0.020; 0.028]	0.100	-	-	-	-	-	-	-
M3d: M3a with autoregressive effects	653.667 (550)	0.002	0.996	0.997	0.016 [0.011; 0.021]	0.075	M3a vs. M3d	27.582 (3)	<0.001	-0.005	-0.005	-0.008	-0.027

Notes. χ^2 (*df*): chi-square test of model fit (degrees of freedom). *CFI*: comparative fit index. *TLI*: Tucker-Lewis index. *RMSEA* [90% *CI*]: root mean square error of approximation [90% confidence interval]. *SRMR*: standardized root mean square residual. $\Delta\chi^2$ (*df*): chi-square difference test (degrees of freedom). Δ*CFI*, Δ*TLI*, Δ*RMSEA*, Δ*SRMR*: differences on the *CFI*, *TLI*, *RMSEA*, and *SRMR* between the two given models. Positive values for the comparisons indicate improvement for the more restrictive model (with larger degrees of freedom) and negative values show decrease in model fit for the more restrictive model. [1]The model was not considered due to statistical problems: the latent variable covariance matrix was not positive definite due to one or more correlations ≥ 1.00.

Supplementary Table 5. Factor loadings, correlations, and internal reliability of the one-factor model of the short version of the Hypersexual Behavior Inventory (HBI-8)

	Wave-specific model (M1a-M1d)				Residual invariance model (M1h)			
**Standardized factor loadings (λ [*SE*])**	**Wave 1**	**Wave 2**	**Wave 3**	**Wave 4**	**Wave 1**	**Wave 2**	**Wave 3**	**Wave 4**
Even though I promised myself I would not repeat a sexual behavior, I find myself returning to it over and over again.	0.91 (0.02)	0.92 (0.02)	0.87 (0.03)	0.76 (0.04)	0.91 (0.01)	0.90 (0.01)	0.87 (0.02)	0.90 (0.02)
I sacrifice things I really want in life in order to be sexual.	0.94 (0.01)	0.88 (0.02)	0.81 (0.03)	0.88 (0.03)	0.91 (0.01)	0.90 (0.02)	0.87 (0.02)	0.90 (0.02)
I turn to sexual activities when I experience unpleasant feelings (e.g. frustration, sadness, anger).	0.93 (0.01)	0.90 (0.02)	0.90 (0.02)	0.91 (0.02)	0.93 (0.01)	0.92 (0.01)	0.90 (0.02)	0.92 (0.01)
When I feel restless, I turn to sex in order to soothe myself.	0.93 (0.01)	0.94 (0.02)	0.87 (0.03)	0.94 (0.02)	0.93 (0.01)	0.92 (0.01)	0.90 (0.02)	0.92 (0.01)
My sexual thoughts and fantasies distract me from accomplishing important tasks.	0.94 (0.01)	0.95 (0.01)	0.91 (0.02)	0.97 (0.01)	0.96 (0.01)	0.95 (0.01)	0.93 (0.01)	0.95 (0.01)
Even though my sexual behavior is irresponsible or reckless, I find it difficult to stop.	0.95 (0.01)	0.91 (0.02)	0.92 (0.02)	0.96 (0.01)	0.95 (0.01)	0.94 (0.01)	0.93 (0.01)	0.94 (0.01)
My sexual cravings and desires feel stronger than my self-discipline.	0.96 (0.01)	0.92 (0.02)	0.94 (0.02)	0.95 (0.02)	0.95 (0.01)	0.94 (0.01)	0.92 (0.01)	0.94 (0.01)
I use sex as a way to try and help myself deal with my problems.	0.95 (0.01)	0.92 (0.02)	0.96 (0.01)	0.97 (0.01)	0.95 (0.01)	0.94 (0.01)	0.93 (0.01)	0.94 (0.01)
Internal consistency (McDonald's ω)	0.98	0.98	0.97	0.98	0.98	0.98	0.97	0.98
Inter-factor correlation (r [*SE*])								
Wave 1					-			
Wave 2					0.60 (0.05)	-		
Wave 3					0.52 (0.05)	0.70 (0.04)	-	
Wave 4					0.09 (0.06)	0.34 (0.07)	0.51 (0.06)	-

Notes. λ [*SE*]: standardized factor loadings (standard error). r [*SE*]: inter-factor correlation (standard error). Except for the inter-factor correlation between waves 1 and 4 ($p = 0.108$), all factor loadings and correlations are significant at $p < 0.001$ level.

Supplementary Table 6. Factor loadings, reliability, consistency and occasion-specificity of each Hypersexual Behavior Inventory (HBI-8) item in the LST model with one trait factor, without method factors and autoregressive effects (M3a model).

Item	Wave	Factor loadings		Reliability (R^2)	Consistency		Occasion-specificity	
		Trait factor *λ* (*SE*)	State residual factors *λ* (*SE*)		*Con* (*SE*)	%	*Spe* (*SE*)	%
1. Even though I promised myself I would not repeat a sexual behavior, I find myself returning to it over and over again	1	0.65 (0.05)	0.67 (0.05)	0.87 (0.01)	0.42 (0.06)	48%	0.45 (0.07)	52%
	2	0.77 (0.03)	0.47 (0.05)	0.82 (0.02)	0.59 (0.05)	72%	0.23 (0.05)	28%
	3	0.79 (0.03)	0.42 (0.06)	0.80 (0.02)	0.63 (0.05)	78%	0.18 (0.05)	22%
	4	0.53 (0.04)	0.79 (0.04)	0.91 (0.02)	0.29 (0.05)	31%	0.63 (0.06)	69%
2. I sacrifice things I really want in life in order to be sexual.	1	0.65 (0.04)	0.66 (0.04)	0.87 (0.01)	0.43 (0.05)	49%	0.44 (0.05)	51%
	2	0.77 (0.03)	0.47 (0.05)	0.81 (0.02)	0.60 (0.04)	73%	0.22 (0.05)	27%
	3	0.80 (0.03)	0.41 (0.05)	0.80 (0.02)	0.63 (0.04)	79%	0.17 (0.04)	21%
	4	0.54 (0.05)	0.78 (0.04)	0.91 (0.02)	0.29 (0.05)	32%	0.61 (0.07)	68%
3. I turn to sexual activities when I experience unpleasant feelings (e.g., frustration, sadness, anger).	1	0.62 (0.03)	0.70 (0.03)	0.88 (0.01)	0.39 (0.04)	44%	0.49 (0.04)	56%
	2	0.75 (0.03)	0.50 (0.05)	0.82 (0.02)	0.57 (0.04)	69%	0.25 (0.05)	31%
	3	0.78 (0.03)	0.45 (0.05)	0.81 (0.02)	0.61 (0.04)	75%	0.20 (0.04)	25%
	4	0.51 (0.04)	0.81 (0.04)	0.92 (0.02)	0.26 (0.05)	28%	0.66 (0.06)	72%
4. When I feel restless, I turn to sex in order to soothe myself.	1	0.59 (0.03)	0.74 (0.03)	0.89 (0.01)	0.34 (0.04)	39%	0.55 (0.05)	61%
	2	0.73 (0.03)	0.55 (0.05)	0.83 (0.02)	0.53 (0.05)	64%	0.30 (0.05)	36%
	3	0.76 (0.03)	0.49 (0.05)	0.82 (0.02)	0.58 (0.05)	71%	0.24 (0.05)	29%
	4	0.47 (0.05)	0.84 (0.03)	0.93 (0.02)	0.22 (0.04)	24%	0.71 (0.06)	77%
5. My sexual thoughts and fantasies distract me from accomplishing important tasks.	1	0.60 (0.04)	0.72 (0.04)	0.89 (0.01)	0.37 (0.05)	41%	0.52 (0.05)	59%
	2	0.74 (0.03)	0.53 (0.05)	0.83 (0.02)	0.55 (0.05)	67%	0.28 (0.05)	33%
	3	0.77 (0.03)	0.47 (0.05)	0.81 (0.02)	0.59 (0.05)	73%	0.22 (0.05)	27%
	4	0.49 (0.05)	0.83 (0.04)	0.93 (0.02)	0.24 (0.05)	26%	0.69 (0.06)	74%
6. Even though my sexual behavior is irresponsible or reckless, I find it difficult to stop.	1	0.61 (0.04)	0.71 (0.04)	0.88 (0.01)	0.38 (0.05)	43%	0.51 (0.06)	57%
	2	0.75 (0.03)	0.52 (0.05)	0.83 (0.02)	0.56 (0.04)	68%	0.27 (0.05)	32%
	3	0.78 (0.03)	0.46 (0.05)	0.81 (0.02)	0.60 (0.05)	74%	0.21 (0.05)	26%
	4	0.50 (0.05)	0.82 (0.04)	0.92 (0.02)	0.25 (0.05)	27%	0.68 (0.06)	73%
7. My sexual cravings and desires feel stronger than my self-discipline.	1	0.56 (0.04)	0.77 (0.04)	0.90 (0.01)	0.32 (0.05)	35%	0.59 (0.05)	65%
	2	0.71 (0.03)	0.58 (0.05)	0.84 (0.02)	0.51 (0.05)	60%	0.33 (0.05)	40%
	3	0.75 (0.03)	0.52 (0.05)	0.83 (0.02)	0.56 (0.05)	67%	0.27 (0.06)	33%
	4	0.44 (0.04)	0.86 (0.03)	0.94 (0.01)	0.20 (0.04)	21%	0.74 (0.05)	79%

Item	Wave	Factor loadings		Reliability (R^2)	Consistency		Occasion-specificity	
		**Trait factor λ (*SE*)**	**State residual factors λ (*SE*)**		***Con* (*SE*)**	**%**	***Spe* (*SE*)**	**%**
8. I use sex as a way to try to help myself deal with my problems.	1	0.55 (0.04)	0.78 (0.03)	0.91 (0.01)	0.30 (0.04)	34%	0.60 (0.05)	66%
	2	0.71 (0.03)	0.59 (0.05)	0.84 (0.02)	0.50 (0.05)	59%	0.35 (0.06)	41%
	3	0.74 (0.04)	0.53 (0.05)	0.83 (0.02)	0.55 (0.05)	66%	0.28 (0.06)	34%
	4	0.43 (0.04)	0.87 (0.03)	0.94 (0.01)	0.19 (0.04)	20%	0.76 (0.05)	80%

Notes. λ (*SE*): standardized factor loading (standard error). *Con* (*SE*): standardized consistency estimate (standard error). *Spe* (*SE*): standardized occasion-specificity estimate (standard error). Standardized consistency and occasion-specificity estimates might not add up to the reliability estimate due to rounding. Percentage values (%) indicate the proportion of variance in true state scores due to consistency (i.e., stable, and time-invariant trait-like effects) and occasion-specificity (i.e., time-varying state-like effects). All parameters are significant at least p <0.001 level.

Supplementary Table 7. Sensitivity analysis: comparison of individuals included in and excluded from the final sample on socio-demographics in wave 1

	Group 1: Final sample	Group 2: Participation and continuous sexual activity in all four waves, but exclusion due to missing values on the HBI-8	Group 3: Participation in all four waves, but exclusion due to the absence of continuous sexual activity and missing values on the HBI-8	Group 4: Exclusion due to other reasons (no participation in all four waves, or no information on continuous sexual activity)	Test statistic and effect size
Age *M* (*SD*)	27.03 (4.77)	27.23 (4.65)	26.60 (4.96)	27.01 (4.80)	F=0.96 η^2=0.00
Gender *N* (*%*)					χ^2=92.08*** V=0.15
Females	281 (39.91%)	576 (57.14%)	112 (78.32%)	1037 (50.98%)	
Males	423 (60.09%)	432 (42.86%)	31 (21.68%)	997 (49.02%)	
Educational attainment *N* (*%*)					χ^2=157.85*** V=0.12
Primary education or less	38 (5.39%)	5 (0.50%)	1 (0.70%)	54 (2.67%)	
Vocational school	130 (18.44%)	289 (28.73%)	18 (12.59%)	335 (16.58%)	
High school graduation	351 (49.79%)	557 (55.37%)	75 (52.45%)	1033 (51.11%)	
Tertiary education or higher	186 (26.38%)	155 (15.41%)	49 (34.27%)	599 (29.64%)	
Working status *N* (*%*)					χ^2=90.77*** V=0.15
Unemployed	94 (13.56%)	86 (8.57%)	42 (29.58%)	409 (20.47%)	
Working	599 (86.44%)	917 (91.43%)	100 (70.42%)	1589 (79.53%)	
Cohabiting romantic relationship *N* (*%*)					χ^2=156.55*** V=0.20
Absence	343 (49.28%)	352 (35.13%)	108 (76.06%)	1097 (56.11%)	
Presence	353 (50.72%)	650 (64.87%)	34 (23.94%)	858 (43.89%)	

Notes. HBI-8: Short version of the Hypersexual Behavior Inventory. *F*: omnibus F-statistic for one-way analysis of variance (ANOVA). η^2: eta squared effect size estimate. χ^2: Pearson's chi-square test. *V*: Cramer's V effect size index. *M (SD):* mean (standard deviation). *N (%)*: number of participants (within-group proportion). All variables were measured in wave 1. Cross-sectional, wave 1-specific weighting was applied for each comparison. Level of significance: *p < 0.050; **p < 0.010; ***p < 0.001.

Supplementary Table 8. Sensitivity analyses: comparisons in terms of the total score of the eight-item Hypersexual Behavior Inventory (HBI-8)

	Group 1: Final sample	Group 2: Participation and continuous sexual activity in all four waves, but exclusion due to missing values on the HBI-8	Group 3: Participation in all four waves, but exclusion due to the absence of continuous sexual activity and missing values on the HBI-8	Group 4: Exclusion due to other reasons (no participation in all four waves, or no information on continuous sexual activity)	*F*	η^2	Significant (p<.05) post-hoc comparisons
	M (SD)	***M (SD)***	***M (SD)***	***M (SD)***			
W1 HBI-8 total score	10.37 (5.07)	10.42 (4.08)	9.90 (3.99)	9.59 (3.96)	10.54***	0.01	1 > 4: *d*=0.18**<br>2 > 4: *d*=0.21***
W2 HBI-8 total score	10.00 (4.78)	8.39 (1.35)	9.47 (3.65)	8.99 (3.31)	29.80***	0.03	1 > 2: *d*=0.49***<br>3 > 2: *d*=0.59**<br>4 > 2: *d*=0.24***<br>1 > 4: *d*=0.25***
W3 HBI-8 total score	9.61 (3.84)	8.57 (2.00)	10.90 (5.97)	8.70 (2.82)	17.34***	0.03	1 > 2: *d*=0.28*<br>3 > 2: *d*=0.44**<br>1 > 4: *d*=0.28***<br>3 > 4: *d*=0.67**
W4 HBI-8 total score	8.77 (2.58)	9.47 (4.14)	9.09 (2.87)	8.52 (1.92)	12.73***	0.02	2 > 1: *d*=0.20***<br>2 > 4: *d*=0.28***

Notes. W1: Wave 1. W2: Wave 2. W3: Wave 3. W4: Wave 4. Group sizes: Group 1 – N_{W1}=703, N_{W2}=647, N_{W3}=677, N_{W4}=773; Group 2 – N_{W1}=999, N_{W2}=862, N_{W3}=36, N_{W4}=894; Group 3 – N_{W1}=135, N_{W2}=135, N_{W3}=105, N_{W4}=162; Group 4 – N_{W1}=1728, N_{W2}=909, N_{W3}=856, N_{W4}=584. *F*: omnibus F-statistic for one-way analysis of variance (ANOVA). η^2: eta squared effect size estimate. *d*: Cohen's d effect size estimate. *M (SD):* mean (standard deviation). Cross-sectional, wave-specific weighting were applied for each comparison. Games-Howell test was used for post-hoc comparisons. Level of significance: *p < 0.050; **p < 0.010; ***p < 0.001.

Supplementary Table 9. Sensitivity analysis: descriptive statistics and pairwise correlations between the eight-item Hypersexual Behavior Inventory (HBI-8) scores in Waves 1, 2 and 4 (W1-W4)

	**1.**	**2.**	**3.**
1. W1 HBI-8 total score	-		
2. W2 HBI-8 total score	0.31***	-	
3. W4 HBI-8 total score	0.03	0.07**	-
M (SD)	10.43 (4.63)	9.37 (3.87)	9.06 (3.34)
Min – Max	8 – 40	8 – 40	8 – 40
Cronbach's α	0.92	0.93	0.94

Notes. N_{Total}=1634 (i.e., considering only the first and second inclusion criteria). W1, W2, W4: scores from waves 1, 2, and 4, respectively. Wave 3 data is not reported, as missing values in wave 3 were assumed to follow a non-random/systematic pattern. *M* (*SD*): mean (standard deviation). Min – Max: minimum and maximum values. Level of significance: *p < 0.05; **p < 0.01; *** p < 0.001.

Supplementary Table 10. Sensitivity analysis: latent growth modelling based on the total score of the Hypersexual Behavior Inventory (HBI-8) across waves 1, 2 and 4

	Intercept	Linear slope	Quadratic slope	Correlation between intercept and linear slope	Correlation between intercept and quadratic slope	Correlation between linear and quadratic slopes
M (*SE*)	10.43 (0.13)***	-1.38 (0.20)***	0.31 (0.06)***	-	-	-
σ^2 (*SE*)	21.39 (1.85)***	49.62 (3.95)***	4.67 (0.40)***	-	-	-
r (*SE*)	-	-	-	-0.63 (0.05)***	0.46 (0.06)***	-0.97 (0.00)***
**Effect size for changes across waves (*d*)**						
Wave 1 – Wave 2	0.18					
Wave 1 – Wave 4	0.18					
Wave 2 – Wave 4	0.05					

Notes. N_{Total}=1634 (i.e., considering only the first and second inclusion criteria). The analysis included HBI-8 scores from waves 1, 2, and 4, while wave 3 data were excluded, as missing values in wave 3 were assumed to follow a non-random/systematic pattern. *M* (*SE*): mean (standard error). σ^2 (*SE*): variance (standard error). *r* (*SE*): standardized covariance (standard error). *d*: repeated measures and pooled Cohen's d controlling for the intercorrelation across measurements. Residual variances of HBI-8 scores in waves 1, 2 and 4 were fixed at 0. The estimated latent growth model was saturated (*df*=0), thus its model fit was not evaluated. Level of significance: *$p < 0.050$; **$p < 0.010$; ***$p < 0.001$.

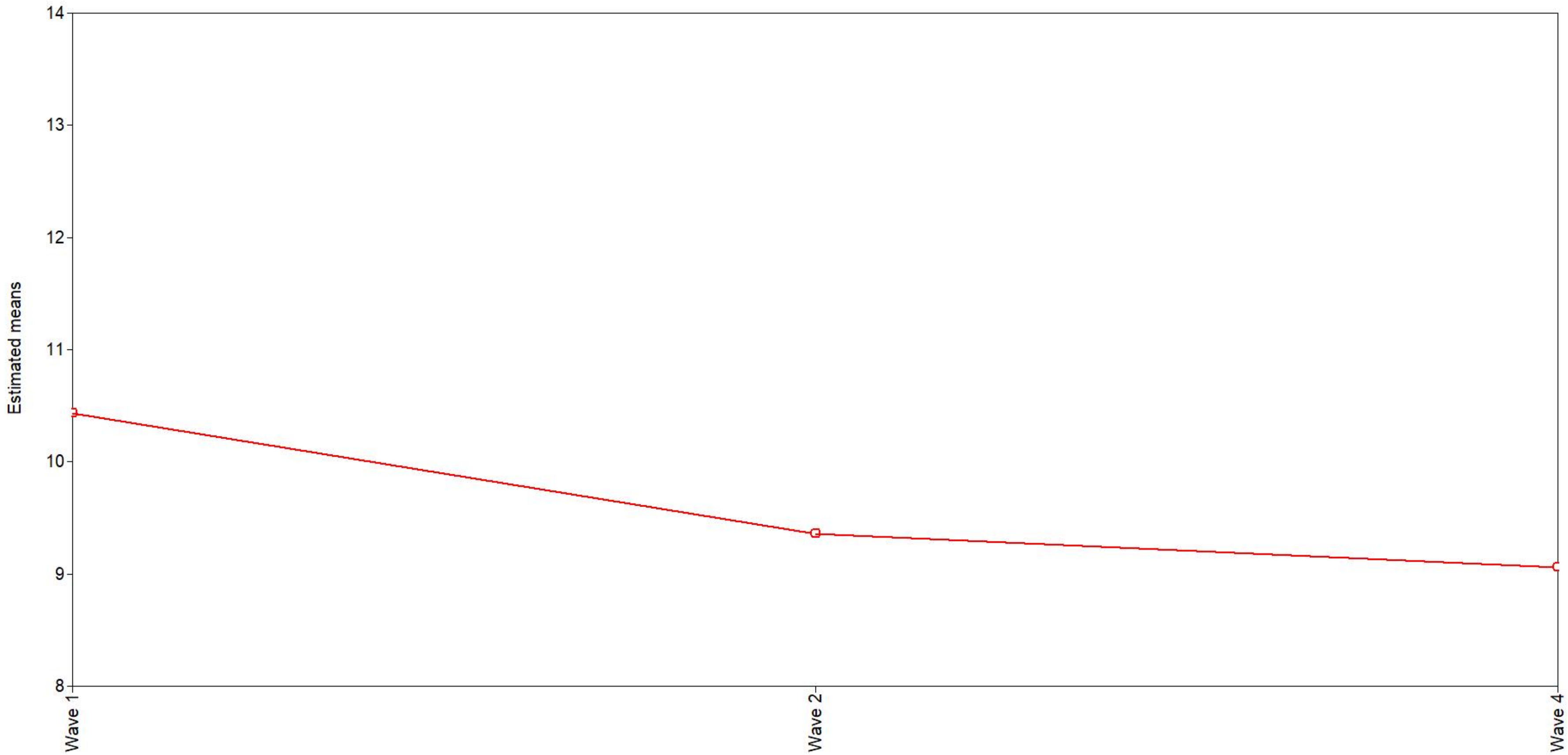

Supplementary Fig. 5. Estimated means of the total score of the of the short version of the Hypersexual Behavior Inventory (HBI-8) based on latent growth modelling across waves 1, 2 and 4.

Notes. N_{Total}=1634 (i.e., considering only the first and second inclusion criteria). The analysis included HBI-8 scores from waves 1, 2, and 4, while wave 3 data were excluded, as missing values in wave 3 were assumed to follow a non-random/systematic pattern.

Supplementary Table 11. Sensitivity analysis: model fit indices of the longitudinal invariance models related to the one-factor model and the latent state-trait (LST) model without method factors and with one trait factor.

	χ^2 (*df*)	*p*	*CFI*	*TLI*	*RMSEA* [90% *CI*]	*SRMR*	Comparison	$\Delta\chi^2$ (*df*)	*p*	Δ*CFI*	Δ*TLI*	Δ*RMSEA*	Δ*SRMR*
**One-factor model (M1)**													
M1a: Wave 1	277.490 (20)	< 0.001	0.984	0.977	0.085 [0.076; 0.094]	0.033	-	-	-	-	-	-	-
M1b: Wave 2	46.652 (20)	< 0.001	0.998	0.997	0.027 [0.017;0.038]	0.015	-	-	-	-	-	-	-
M1c: Wave 4	132.918 (20)	< 0.001	0.997	0.996	0.057 [0.048; 0.066]	0.021	-	-	-	-	-	-	-
M1d: Configural invariance	461.680 (249)	< 0.001	0.996	0.995	0.022 [0.019; 0.025]	0.058	-	-	-	-	-	-	-
M1e: Metric invariance	498.932 (263)	< 0.001	0.995	0.995	0.022 [0.019; 0.025]	0.058	M1d vs. M1e	73.477 (14)	< 0.001	- 0.001	0.000	0.000	0.000
M1f: Scalar invariance	595.981 (293)	< 0.001	0.994	0.994	0.024 [0.021; 0.027]	0.058	M1e vs. M1f	149.676 (30)	< 0.001	- 0.001	- 0.001	-0.002	0.000
M1g: Residual invariance	709.379 (309)	< 0.001	0.992	0.993	0.027 [0.024; 0.029]	0.060	M1f vs. M1g	148.245 (16)	< 0.001	- 0.002	- 0.001	-0.003	-0.002
**Latent state-trait (LST) model: one trait factor without method factors (M2)**													
M2a: without autoregressive effects	763.939 (311)	< 0.001	0.991	0.992	0.028 [0.026; 0.031]	0.095	-	-	-	-	-	-	-
M2b: with autoregressive effects	707.822 (309)	< 0.001	0.992	0.993	0.027 [0.024; 0.029]	0.062	M2a vs. M2b	27.408 (2)	< 0.001	- 0.001	- 0.001	-0.001	-0.033

Notes. N_{Total}=1634 (i.e., considering only the first and second inclusion criteria). The analysis included HBI-8 scores from waves 1, 2, and 4, while wave 3 data were excluded, as missing values in wave 3 were assumed to follow a non-random/systematic pattern. χ^2 (*df*): chi-square test of model fit (degrees of freedom). *CFI*: comparative fit index. *TLI*: Tucker-Lewis index. *RMSEA* [90% *CI*]: root mean square error of approximation [90% confidence interval]. *SRMR*: standardized root mean square residual. $\Delta\chi^2$ (*df*): chi-square difference test (degrees of freedom). Δ*CFI*, Δ*TLI*, Δ*RMSEA*, Δ*SRMR*: differences on the *CFI*, *TLI*, *RMSEA*, and *SRMR* between the two given models. Positive values for the comparisons indicate improvement for the more restrictive model (with larger degrees of freedom) and negative values show decrease in model fit for the more restrictive model.

Supplementary Table 12. Sensitivity analysis: factor loadings, reliability, consistency and occasion-specificity of each Hypersexual Behavior Inventory (HBI-8) item

Item	Wave	Factor loadings		Reliability (R^2)	Consistency		Occasion-specificity	
		Trait factor *λ* (*SE*)	State residual factors *λ* (*SE*)		*Con* (*SE*)	%	*Spe* (*SE*)	%
1. Even though I promised myself I would not repeat a sexual behavior, I find myself returning to it over and over again	1	0.14 (0.38)	0.84 (0.06)***	0.72 (0.02)***	0.02 (0.11)	3%	0.70 (0.11)***	97%
	2	0.12 (0.33)	0.88 (0.05)***	0.79 (0.02)***	0.02 (0.08)	2%	0.78 (0.08)***	98%
	4	0.11 (0.28)	0.91 (0.04)***	0.84 (0.02)***	0.01 (0.06)	1%	0.83 (0.07)***	99%
2. I sacrifice things I really want in life in order to be sexual.	1	0.13 (0.35)	0.87 (0.06)***	0.77 (0.02)***	0.02 (0.09)	2%	0.75 (0.09)***	98%
	2	0.11 (0.30)	0.90 (0.04)***	0.83 (0.02)***	0.01 (0.07)	2%	0.82 (0.07)***	99%
	4	0.10 (0.26)	0.93 (0.03)***	0.87 (0.02)***	0.01 (0.05)	1%	0.86 (0.06)***	99%
3. I turn to sexual activities when I experience unpleasant feelings (e.g., frustration, sadness, anger).	1	0.14 (0.36)	0.86 (0.06)***	0.75 (0.02)***	0.02 (0.10)	2%	0.74 (0.10)***	98%
	2	0.12 (0.30)	0.90 (0.04)***	0.82 (0.02)***	0.01 (0.07)	2%	0.81 (0.07)***	98%
	4	0.10 (0.26)	0.93 (0.03)***	0.87 (0.02)***	0.01 (0.05)	1%	0.86 (0.06)***	99%
4. When I feel restless, I turn to sex in order to soothe myself.	1	0.12 (0.32)	0.89 (0.05)***	0.80 (0.02)***	0.02 (0.08)	2%	0.78 (0.08)***	98%
	2	0.10 (0.27)	0.92 (0.03)***	0.86 (0.02)***	0.01 (0.06)	1%	0.85 (0.06)***	99%
	4	0.09 (0.24)	0.94 (0.02)***	0.89 (0.01)***	0.01 (0.04)	1%	0.89 (0.04)***	99%
5. My sexual thoughts and fantasies distract me from accomplishing important tasks.	1	0.11 (0.29)	0.91 (0.04)***	0.84 (0.02)***	0.01 (0.06)	1%	0.83 (0.07)***	99%
	2	0.09 (0.24)	0.94 (0.03)***	0.89 (0.02)***	0.01 (0.04)	1%	0.88 (0.05)***	99%

Item	Wave	Factor loadings: Trait factor λ (*SE*)	Factor loadings: State residual factors λ (*SE*)	Reliability (R^2)	Consistency: *Con* (*SE*)	Consistency: %	Occasion-specificity: *Spe* (*SE*)	Occasion-specificity: %
	4	0.08 (0.21)	0.95 (0.02)***	0.92 (0.01)***	0.01 (0.03)	1%	0.91 (0.03)***	99%
6. Even though my sexual behavior is irresponsible or reckless, I find it difficult to stop.	1	0.11 (0.28)	0.92 (0.03)***	0.85 (0.02)***	0.01 (0.06)	1%	0.84 (0.06)***	99%
	2	0.09 (0.23)	0.94 (0.02)***	0.89 (0.02)***	0.01 (0.04)	1%	0.89 (0.04)***	99%
	4	0.08 (0.20)	0.96 (0.02)***	0.92 (0.01)***	0.01 (0.03)	1%	0.92 (0.03)***	99%
7. My sexual cravings and desires feel stronger than my self-discipline.	1	0.12 (0.31)	0.89 (0.04)***	0.81 (0.02)***	0.01 (0.07)	2%	0.80 (0.08)***	98%
	2	0.10 (0.26)	0.93 (0.03)***	0.87 (0.02)***	0.01 (0.05)	1%	0.86 (0.06)***	99%
	4	0.09 (0.23)	0.95 (0.02)***	0.90 (0.01)***	0.01 (0.04)	1%	0.89 (0.04)***	99%
8. I use sex as a way to try to help myself deal with my problems.	1	0.12 (0.31)	0.89 (0.04)***	0.81 (0.02)***	0.01 (0.07)	2%	0.80 (0.08)***	98%
	2	0.10 (0.26)	0.93 (0.03)***	0.87 (0.02)***	0.01 (0.05)	1%	0.86 (0.06)***	99%
	4	0.09 (0.23)	0.95 (0.02)***	0.90 (0.01)***	0.01 (0.04)	1%	0.89 (0.04)***	99%

Notes. Notes. N_{Total}=1634 (i.e., considering only the first and second inclusion criteria). The analysis included HBI-8 scores from waves 1, 2, and 4, while wave 3 data were excluded, as missing values in wave 3 were assumed to follow a non-random/systematic pattern. λ (*SE*): standardized factor loading (standard error). *Con* (*SE*): standardized consistency estimate (standard error). *Spe* (*SE*): standardized occasion-specificity estimate (standard error). Standardized consistency and occasion-specificity estimates might not add up to the reliability estimate due to rounding. Percentage values (%) indicate the proportion of variance in true state scores due to consistency (i.e., stable, and time-invariant trait-like effects) and occasion-specificity (i.e., time-varying state-like effects). Standardized autoregressive effects between consecutive state residual factors: waves 1 and 2 – *β* (*SE*) = 0.47 (0.07), $p < 0.001$; waves 2 and 4 – *β* (*SE*) = 0.22 (0.08), $p = 0.007$. Level of significance: *$p < 0.050$; **$p < 0.010$; *** $p < 0.001$.

References

Ballester-Arnal, R., Castro-Calvo, J., Gil-Julia, B., Giménez-García, C., & Gil-Llario, M. D. (2019). A Validation Study of the Spanish Version of the Hypersexual Behavior Inventory (HBI): Paper-and-Pencil Versus Online Administration. *Journal of Sex & Marital Therapy*, *45*(4), 283–302. https://doi.org/10.1080/0092623X.2018.1518886

Ballester-Arnal, R., Gómez-Martínez, S., Llario, M. D.-G., & Salmerón-Sánchez, P. (2013). Sexual Compulsivity Scale: Adaptation and Validation in the Spanish Population. *Journal of Sex & Marital Therapy*, *39*(6), 526–540. https://doi.org/10.1080/0092623X.2012.665816

Bőthe, B., Kovács, M., Tóth-Király, I., Reid, R. C., Griffiths, M. D., Orosz, G., & Demetrovics, Z. (2019). The Psychometric Properties of the Hypersexual Behavior Inventory Using a Large-Scale Nonclinical Sample. *The Journal of Sex Research*, *56*(2), 180–190. https://doi.org/10.1080/00224499.2018.1494262

Bőthe, B., Vaillancourt-Morel, M.-P., & Bergeron, S. (2021). Hypersexuality in Mixed-Sex Couples: A Dyadic Longitudinal Study. *Archives of Sexual Behavior*, *50*(5), 2139–2150. https://doi.org/10.1007/s10508-021-01959-0

Chen, F. F. (2007). Sensitivity of Goodness of Fit Indexes to Lack of Measurement Invariance. *Structural Equation Modeling: A Multidisciplinary Journal*, *14*(3), 464–504. https://doi.org/10.1080/10705510701301834

Deng, J., Li, T., Wang, J., & Teng, L. (2021). Longitudinal influence of COVID-19-related stress on sexual compulsivity symptoms in Chinese undergraduates. *BMC Psychiatry*, *21*(1), 376. https://doi.org/10.1186/s12888-021-03369-x

Efrati, Y. (2024). Parental practices as predictors of adolescents' compulsive sexual behavior: A 6-month prospective study. *European Child & Adolescent Psychiatry*, *33*(1), 241–253. https://doi.org/10.1007/s00787-023-02155-2

Geiser, C., & Lockhart, G. (2012). A comparison of four approaches to account for method effects in latent state–trait analyses. *Psychological Methods*, *17*(2), 255–283. https://doi.org/10.1037/a0026977

Gomez, R., Brown, T., & Stavropoulos, V. (2024). The Bergen–Yale Sexual Addiction Scale (BYSAS): Longitudinal Measurement Invariance Across a Two-Year Interval. *Psychiatric Quarterly*, *95*(4), 561–577. https://doi.org/10.1007/s11126-024-10087-6

Grassi, G., Moradei, C., & Cecchelli, C. (2024). Long-term changes on behavioral addictions symptoms among adults with attention deficit hyperactivity disorder treated with methylphenidate. *Journal of Behavioral Addictions*, *13*(4), 904–912. https://doi.org/10.1556/2006.2024.00060

Grubbs, J. B., Reid, R. C., Bőthe, B., Demetrovics, Z., Coleman, E., Gleason, N., Miner, M. H., Fuss, J., Klein, V., Lewczuk, K., Gola, M., Fernandez, D. P., Fernandez, E. F., Carnes, S., Lew-Starowicz, M., Kingston, D., & Kraus, S. W. (2023). Assessing compulsive sexual behavior disorder: The development and international validation of the compulsive sexual behavior disorder-diagnostic inventory (CSBD-DI). *Journal of Behavioral Addictions*, *12*(1), 242–260. https://doi.org/10.1556/2006.2023.00005

Koós, M., Demetrovics, Z., Griffiths, M. D., & Bőthe, B. (2022). No Significant Changes in Addictive and Problematic Behaviors During the COVID-19 Pandemic and Related Lockdowns: A Three-Wave Longitudinal Study. *Frontiers in Psychology*, *13*, 837315. https://doi.org/10.3389/fpsyg.2022.837315

Kürbitz, L. I., Wiessner, C., Schoon, W., Briken, P., Schöttle, D., & Schröder, J. (2022). Gender differences in the association of psychological distress and sexual compulsivity before and during the COVID-19 pandemic. *Journal of Behavioral Addictions*. https://doi.org/10.1556/2006.2022.00046

Liu, Y., Millsap, R. E., West, S. G., Tein, J.-Y., Tanaka, R., & Grimm, K. J. (2017). Testing measurement invariance in longitudinal data with ordered-categorical measures. *Psychological Methods*, *22*(3), 486–506. https://doi.org/10.1037/met0000075

Muthén, L. K., & Muthén, B. O. (2017). *Mplus User's Guide.* (Eight Edition). Muthén & Muthén.

Reid, R. C., Carpenter, B. N., Hook, J. N., Garos, S., Manning, J. C., Gilliland, R., Cooper, E. B., McKittrick, H., Davtian, M., & Fong, T. (2012). Report of Findings in a DSM-5 Field Trial for Hypersexual Disorder. *The Journal of Sexual Medicine*, *9*(11), 2868–2877. https://doi.org/10.1111/j.1743-6109.2012.02936.x

Reid, R. C., Garos, S., & Carpenter, B. N. (2011). Reliability, Validity, and Psychometric Development of the Hypersexual Behavior Inventory in an Outpatient Sample of Men. *Sexual Addiction & Compulsivity*, *18*(1), 30–51. https://doi.org/10.1080/10720162.2011.555709

Rosansky, J. A., Borgogna, N. C., Kraus, S. W., & Grubbs, J. B. (2022). Cross-Sectional and Longitudinal Associations Between Posttraumatic Stress Symptoms and Hypersexual Behaviors Among Individuals Who have Gambled in Their Lifetimes. *The Journal of Sexual Medicine*, *19*(12), 1813–1823. https://doi.org/10.1016/j.jsxm.2022.09.003

Smith, P., Potenza, M., Mazure, C., McKee, S., Park, C., & Hoff, R. (2014). Compulsive sexual behavior among male military veterans: Prevalence and associated clinical factors. *Journal of Behavioral Addictions*, *3*(4), 214–222. https://doi.org/10.1556/JBA.3.2014.4.2

Thompson, M. P., Kingree, J. B., Zinzow, H., & Swartout, K. (2015). Time-Varying Risk Factors and Sexual Aggression Perpetration Among Male College Students. *Journal of Adolescent Health*, *57*(6), 637–642. https://doi.org/10.1016/j.jadohealth.2015.08.015
